# Supplementary material for: Multilayer network analysis across cortical depths in 7-T resting-state fMRI
Source: Netw Neurosci. 2025 Apr 30;9(2):475–503. doi: 10.1162/netn_a_00436 (PMC12151305; doi:10.1162/netn_a_00436)
Supplement: Supplementary file 1 [file netn-9-2-475-s001.pdf]

Multilayer Network Analysis across Cortical Depths in Resting-State 7T fMRI

Supplement

Table S1. List of node identifier numbers, region of interest (ROI) short and long names, and their associated brain region. The data in this table is adapted from *Destrieux et al.* (Destrieux et al., 2010; Fischl et al., 2004).

L. No.	R. No.	Brain Region	ROI Short Name	ROI Long Name
1	75	Frontal	G_and_S_frontomargin	Fronto-marginal gyrus (of Wernicke) and sulcus
2	76	Occipital	G_and_S_occipital_inf	Inferior occipital gyrus (O3) and sulcus
3	77	Frontal	G_and_S_paracentral	Paracentral lobule and sulcus
4	78	Frontal	G_and_S_subcentral	Subcentral gyrus (central operculum) and sulci
5	79	Frontal	G_and_S_transv_frontopol	Transverse frontopolar gyri and sulci
6	80	Limbic	G_and_S_cingul-Ant	Anterior part of the cingulate gyrus and sulcus (ACC)
7	81	Limbic	G_and_S_cingul-Mid-Ant	Middle-anterior part of the cingulate gyrus and sulcus (aMCC)
8	82	Limbic	G_and_S_cingul-Mid-Post	Middle-posterior part of the cingulate gyrus and sulcus (pMCC)
9	83	Limbic	G_cingul-Post-dorsal	Posterior-dorsal part of the cingulate gyrus (dPCC)
10	84	Limbic	G_cingul-Post-ventral	Posterior-ventral part of the cingulate gyrus (vPCC, isthmus of the cingulate gyrus)
11	85	Occipital	G_cuneus	Cuneus (O6)
12	86	Frontal	G_front_inf-Opercular	Opercular part of the inferior frontal gyrus
13	87	Frontal	G_front_inf-Orbital	Orbital part of the inferior frontal gyrus
14	88	Frontal	G_front_inf-Triangul	Triangular part of the inferior frontal gyrus
15	89	Frontal	G_front_middle	Middle frontal gyrus (F2)
16	90	Frontal	G_front_sup	Superior frontal gyrus (F1)
17	91	Limbic	G_Ins_lg_and_S_cent_ins	Long insular gyrus and central sulcus of the insula
18	92	Limbic	G_insular_short	Short insular gyri
19	93	Occipital	G_occipital_middle	Middle occipital gyrus (O2, lateral occipital gyrus)
20	94	Occipital	G_occipital_sup	Superior occipital gyrus (O1)
21	95	Temporal	G_oc-temp_lat-fusifor	Lateral occipito-temporal gyrus (fusiform gyrus, O4-T4)
22	96	Temporal	G_oc-temp_med-Lingual	Lingual gyrus, ligual part of the medial occipito-temporal gyrus, (O5)
23	97	Temporal	G_oc-temp_med-Parahip	Parahippocampal gyrus, parahippocampal part of the medial occipito-temporal gyrus, (T5)
24	98	Frontal	G_orbital	Orbital gyri
25	99	Parietal	G_pariet_inf-Angular	Angular gyrus
26	100	Parietal	G_pariet_inf-Supramar	Supramarginal gyrus

27	101	Parietal	G_parietal_sup	Superior parietal lobule (lateral part of P1)
28	102	Parietal	G_postcentral	Postcentral gyrus
29	103	Frontal	G_precentral	Precentral gyrus
30	104	Parietal	G_precuneus	Precuneus (medial part of P1)
31	105	Frontal	G_rectus	Straight gyrus, Gyrus rectus
32	106	Limbic	G_subcallosal	Subcallosal area, subcallosal gyrus
33	107	Temporal	G_temp_sup-G_T_transv	Anterior transverse temporal gyrus (of Heschl)
34	108	Temporal	G_temp_sup-Lateral	Lateral aspect of the superior temporal gyrus
35	109	Temporal	G_temp_sup-Plan_polar	Planum polare of the superior temporal gyrus
36	110	Temporal	G_temp_sup-Plan_tempo	Planum temporale or temporal plane of the superior temporal gyrus
37	111	Temporal	G_temporal_inf	Inferior temporal gyrus (T3)
38	112	Temporal	G_temporal_middle	Middle temporal gyrus (T2)
39	113	Frontal	Lat_Fis-ant-Horizont	Horizontal ramus of the anterior segment of the lateral sulcus (or fissure)
40	114	Frontal	Lat_Fis-ant-Vertical	Vertical ramus of the anterior segment of the lateral sulcus (or fissure)
41	115	Limbic	Lat_Fis-post	Posterior ramus (or segment) of the lateral sulcus (or fissure)
42	116	Occipital	Pole_occipital	Occipital pole
43	117	Temporal	Pole_temporal	Temporal pole
44	118	Occipital	S_calcarine	Calcarine sulcus
45	119	Frontal	S_central	Central sulcus (Rolando’s fissure)
46	120	Limbic	S_cingul-Marginalis	Marginal branch (or part) of the cingulate sulcus
47	121	Limbic	S_circular_insula_ant	Anterior segment of the circular sulcus of the insula
48	122	Limbic	S_circular_insula_inf	Inferior segment of the circular sulcus of the insula
49	123	Limbic	S_circular_insula_sup	Superior segment of the circular sulcus of the insula
50	124	Temporal	S_collat_transv_ant	Anterior transverse collateral sulcus
51	125	Temporal	S_collat_transv_post	Posterior transverse collateral sulcus
52	126	Frontal	S_front_inf	Inferior frontal sulcus
53	127	Frontal	S_front_middle	Middle frontal sulcus
54	128	Frontal	S_front_sup	Superior frontal sulcus
55	129	Parietal	S_interm_prim-Jensen	Sulcus intermedius primus (of Jensen)
56	130	Parietal	S_intrapariet_and_P_trans	Intraparietal sulcus (interparietal sulcus) and transverse parietal sulci
57	131	Occipital	S_oc_middle_and_Lunatus	Middle occipital sulcus and lunatus sulcus
58	132	Occipital	S_oc_sup_and_transversal	Superior occipital sulcus and transverse occipital sulcus
59	133	Occipital	S_occipital_ant	Anterior occipital sulcus and preoccipital notch (temporo-occipital incisure)

60	134	Occipital	S_oc-temp_lat	Lateral occipito-temporal sulcus
61	135	Temporal	S_oc-temp_med_and_Lingual	Medial occipito-temporal sulcus (collateral sulcus) and lingual sulcus
62	136	Frontal	S_orbital_lateral	Lateral orbital sulcus
63	137	Frontal	S_orbital_med-olfact	Medial orbital sulcus (olfactory sulcus)
64	138	Frontal	S_orbital-H_Shaped	Orbital sulci (H-shaped sulci)
65	139	Parietal	S_parieto_occipital	Parieto-occipital sulcus (or fissure)
66	140	Limbic	S_pericallosal	Pericallosal sulcus (S of corpus callosum)
67	141	Parietal	S_postcentral	Postcentral sulcus
68	142	Frontal	S_precentral-inf-part	Inferior part of the precentral sulcus
69	143	Frontal	S_precentral-sup-part	Superior part of the precentral sulcus
70	144	Frontal	S_suborbital	Suborbital sulcus (sulcus rostrales, supraorbital sulcus)
71	145	Parietal	S_subparietal	Subparietal sulcus
72	146	Temporal	S_temporal_inf	Inferior temporal sulcus
73	147	Temporal	S_temporal_sup	Superior temporal sulcus (parallel sulcus)
74	148	Temporal	S_temporal_transverse	Transverse temporal sulcus

Table S2. Area-under-the-curve (AUC) values for each global measure for layer-by-layer analysis. Data are shown as mean ± standard error of mean (standard deviation). One-way ANOVA was used to compare layers with an FDR correction (alpha = 0.05). Graph density and average degree centrality do not have standard error and standard deviation since they are consistent across all participants due to thresholding. Shaded rows have significant differences between layers. P values shown are false discovery rate corrected (FDR, Benjamini-Hochberg method, alpha = 0.05).
* p < 0.05, ** p < 0.01.

Measures (AUC)	Layer 1 (Superficial)	Layer 2	Layer 3	Layer 4	Layer 5 (Deep)	p value
Modularity	13.33 + 0.52 (2.83)	13.24 + 0.56 (3.08)	12.90 + 0.63 (3.43)	12.27 + 0.68 (3.71)	11.48 + 0.68 (3.71)	0.32
Transitivity	10.81 + 0.32 (1.78)	11.03 + 0.35 (1.90)	11.29 + 0.40 (2.17)	11.47 + 0.44 (2.43)	11.52 + 0.46 (2.51)	0.73
Largest Cluster Size	5167.97 + 36.53 (200.09)	5151.87 + 41.83 (229.11)	5059.57 + 58.99 (323.10)	4928.27 + 78.80 (431.60)	4802.27 + 86.16 (471.93)	0.0024**
Graph Density	7.98	7.98	7.98	7.98	7.98	NaN
Characteristic Path Length	204.42 + 5.30 (29.06)	205.75 + 4.96 (27.19)	207.48 + 4.77 (26.11)	210.86 + 5.40 (29.58)	214.96 + 5.85 (32.02)	0.73
Global Efficiency	8.97 + 0.21 (1.17)	8.99 + 0.21 (1.13)	9.05 + 0.23 (1.23)	9.09 + 0.29 (1.58)	9.07 + 0.31 (1.69)	0.99
Radius	228.37 + 11.40 (62.41)	244.91 + 9.13 (50.00)	249.15 + 9.67 (52.94)	258.64 + 10.85 (59.42)	271.26 + 9.97 (54.62)	0.12
Diameter	556.97 + 17.06 (93.47)	568.48 + 14.95 (81.89)	582.71 + 14.71 (80.57)	608.22 + 17.49 (95.78)	629.05 + 18.98 (103.97)	0.049*
Assortativity	9.19 + 0.47 (2.55)	9.13 + 0.50 (2.72)	8.58 + 0.54 (2.96)	8.11 + 0.56 (3.04)	7.62 + 0.59 (3.21)	0.32
Avg. Degree Centrality	1173.07	1173.07	1173.07	1173.07	1173.07	NaN
Avg. Strength	483.02 + 11.27 (61.70)	485.82 + 10.73 (58.77)	484.59 + 10.61 (58.09)	477.20 + 10.95 (59.98)	465.23 + 10.05 (55.07)	0.73
Avg. Eigenvector Centrality	2.26 + 0.02 (0.10)	2.25 + 0.02 (0.13)	2.22 + 0.03 (0.14)	2.18 + 0.03 (0.14)	2.14 + 0.03 (0.15)	0.028*
Avg. Betweenness Centrality	6000.07 + 138.76 (760.03)	6089.39 + 150.75 (825.71)	5904.46 + 155.92 (853.98)	5596.98 + 194.75 (1066.66)	5270.58 + 207.93 (1138.90)	0.028*
Avg. Clustering Coefficient	9.68 + 0.22 (1.23)	9.73 + 0.22 (1.20)	9.74 + 0.23 (1.23)	9.56 + 0.23 (1.25)	9.27 + 0.21 (1.13)	0.73
Avg. Local Efficiency	11.86 + 0.25 (1.39)	11.88 + 0.24 (1.30)	11.75 + 0.23 (1.29)	11.38 + 0.22 (1.23)	10.91 + 0.19 (1.02)	0.046*
Avg. Participation Coefficient	11.82 + 0.47 (2.55)	11.64 + 0.49 (2.69)	11.05 + 0.58 (3.17)	10.46 + 0.67 (3.70)	10.16 + 0.74 (4.04)	0.34

Table S3. Area-under-the-curve (AUC) values for each significant nodal measure for layer-by-layer analysis. Data are shown as mean

± standard error (standard deviation). One-way ANOVA was used to compare layers. Node long names can be found in Table S1. P values shown are false discovery rate corrected (FDR, Bonferroni-Holm method, alpha = 0.01).
* p < 0.05, ** p < 0.01.

Node	Layer 1 (Superficial)	Layer 2	Layer 3	Layer 4	Layer 5 (Deep)	P value
Degree centrality						
Left G_cingul-Post-ventral (10)	982.70 + 80.68 (441.88)	933.40 + 89.14 (488.26)	729.17 + 80.08 (438.60)	534.27 + 74.18 (406.29)	381.17 + 64.72 (354.47)	< 0.001**
Right G_cingul-Post-ventral (84)	1018.53 + 99.39 (544.36)	956.00 + 88.54 (484.95)	717.40 + 84.96 (465.34)	509.10 + 78.99 (432.63)	352.03 + 68.67 (376.14)	< 0.001**
Right S_circular_insula_sup (123)	1253.10 + 75.06 (411.11)	1370.90 + 83.90 (459.53)	1280.87 + 85.07 (465.93)	903.67 + 94.45 (517.35)	683.17 + 93.22 (510.59)	< 0.001**
Right S_temporal_transverse (148)	916.00 + 100.47 (550.31)	858.67 + 107.08 (586.50)	625.30 + 88.76 (486.14)	451.30 + 86.03 (471.18)	355.03 + 76.07 (416.68)	0.0046**
Strength						
Left G_cingul-Post-ventral (10)	360.76 + 32.89 (180.14)	341.90 + 36.03 (197.36)	256.32 + 31.64 (173.28)	174.36 + 26.57 (145.55)	114.12 + 20.26 (110.96)	< 0.001**
Right G_cingul-Post-ventral (84)	382.55 + 41.50 (227.32)	352.85 + 37.36 (204.62)	254.66 + 34.26 (187.63)	168.14 + 29.52 (161.68)	107.18 + 23.87 (130.72)	< 0.001**
Right S_circular_insula_sup (123)	490.27 + 34.36 (188.18)	537.60 + 36.96 (202.46)	485.49 + 35.65 (195.25)	325.06 + 39.46 (216.13)	234.57 + 36.81 (201.64)	< 0.001**
Right S_temporal_transverse (148)	344.05 + 43.08 (235.98)	320.15 + 44.47 (243.59)	221.59 + 36.52 (200.02)	151.01 + 32.12 (175.90)	114.20 + 26.17 (143.35)	0.0026**
Eigenvector Centrality						
Left G_cingul-Post-ventral (10)	1.66 + 0.23 (1.24)	1.61 + 0.22 (1.20)	1.19 + 0.18 (0.98)	0.78 + 0.14 (0.77)	0.51 + 0.11 (0.60)	0.0016**
Right G_cingul-Post-ventral (84)	1.79 + 0.23 (1.28)	1.66 + 0.20 (1.11)	1.18 + 0.17 (0.96)	0.76 + 0.14 (0.79)	0.48 + 0.11 (0.62)	< 0.001**
Right S_circular_insula_sup (123)	2.52 + 0.26 (1.41)	2.74 + 0.28 (1.55)	2.40 + 0.25 (1.39)	1.59 + 0.24 (1.32)	1.15 + 0.22 (1.20)	0.0035**
Right S_temporal_transverse (148)	1.72 + 0.27 (1.50)	1.50 + 0.26 (1.43)	0.92 + 0.19 (1.06)	0.57 + 0.16 (0.89)	0.43 + 0.15 (0.83)	0.0052**
Betweenness Centrality						
Right G_cingul-Post-ventral (84)	1842.87 + 492.14 (2695.58)	1292.67 + 366.50 (2007.43)	420.93 + 233.78 (1280.47)	142.27 + 65.30 (357.66)	60.87 + 48.72 (266.83)	0.0054**
Right G_front_inf-Opercular (86)	8238.80 + 1148.64 (6291.38)	5349.93 + 730.46 (4000.89)	3726.60 + 582.99 (3193.16)	2879.87 + 514.49 (2817.98)	2465.00 + 567.53 (3108.50)	< 0.001**
Right S_circular_insula_sup (123)	5156.67 + 879.22 (4815.66)	6725.47 + 1273.00 (6972.51)	5025.60 + 796.93 (4364.98)	1866.33 + 463.74 (2539.99)	1106.67 + 304.51 (1667.88)	< 0.001**
Clustering Coefficient						
Right G_cingul-Post-ventral (84)	9.96 + 0.44 (2.41)	10.02 + 0.45 (2.45)	9.04 + 0.62 (3.38)	7.23 + 0.70 (3.82)	5.53 + 0.70 (3.81)	< 0.001**
Right S_temporal_transverse (148)	11.02 + 0.51	9.94 + 0.49 (2.71)	8.63 + 0.61 (3.33)	6.75 + 0.81 (4.41)	6.22 + 0.80 (4.39)	< 0.001**

	(2.80)					
Local Efficiency						
Left G_cingul-Post-ventral (10)	10.98 + 0.57 (3.14)	10.60 + 0.63 (3.45)	9.36 + 0.74 (4.07)	8.03 + 0.74 (4.07)	6.19 + 0.79 (4.31)	0.0013**
Right G_cingul-Post-ventral (84)	11.56 + 0.51 (2.77)	11.42 + 0.53 (2.88)	9.95 + 0.71 (3.88)	7.74 + 0.77 (4.22)	5.80 + 0.76 (4.16)	< 0.001**
Right S_circular_insula_inf (122)	11.64 + 0.44 (2.41)	11.79 + 0.51 (2.80)	10.28 + 0.78 (4.26)	8.54 + 0.86 (4.73)	7.47 + 0.86 (4.72)	0.0043**
Right S_temporal_transverse (148)	12.33 + 0.50 (2.72)	11.14 + 0.52 (2.82)	9.47 + 0.65 (3.55)	7.27 + 0.84 (4.62)	6.61 + 0.84 (4.62)	< 0.001**
Participation Coefficient						
Left G_cingul-Post-ventral (10)	13.99 + 1.07 (5.84)	13.47 + 0.99 (5.44)	11.33 + 1.13 (6.18)	8.96 + 1.14 (6.24)	6.49 + 1.03 (5.65)	< 0.001**
Right G_cingul-Post-ventral (84)	13.21 + 1.22 (6.68)	13.12 + 1.13 (6.20)	10.92 + 1.25 (6.83)	7.99 + 1.27 (6.95)	5.57 + 1.04 (5.71)	0.0013**
Right S_circular_insula_sup (123)	13.12 + 0.81 (4.45)	13.94 + 0.91 (4.98)	12.17 + 1.06 (5.80)	8.24 + 1.18 (6.49)	6.84 + 1.16 (6.37)	< 0.001**

Table S4. Area-under-the-curve (AUC) values for each global measure for within-layer analysis. Data are shown as mean ± standard error (standard deviation). One-way ANOVA was used to compare layers with an FDR correction (alpha = 0.05). Shaded rows have significant differences between layers. P values shown are false discovery rate corrected (FDR, Benjamini-Hochberg method, alpha = 0.05).
* p < 0.05, ** p < 0.01.

Measures (AUC)	Layer 1 (Superficial)	Layer 2	Layer 3	Layer 4	Layer 5 (Deep)	p value
Modularity	12.46 + 0.57 (3.12)	12.57 + 0.59 (3.20)	12.62 + 0.63 (3.47)	12.54 + 0.69 (3.79)	12.53 + 0.72 (3.92)	0.99
Transitivity	6.40 + 0.20 (1.12)	6.48 + 0.23 (1.25)	6.65 + 0.28 (1.52)	6.84 + 0.32 (1.76)	6.93 + 0.35 (1.94)	0.85
Largest Cluster Size	5185.47 + 55.46 (303.78)	5172.47 + 55.98 (306.64)	5085.87 + 57.87 (316.96)	4931.50 + 62.69 (343.35)	4672.17 + 63.20 (346.18)	< 0.001**
Graph Density	9.33 + 0.30 (1.66)	9.00 + 0.19 (1.05)	8.48 + 0.10 (0.55)	7.83 + 0.19 (1.03)	6.84 + 0.28 (1.55)	< 0.001**
Characteristic Path Length	335.95 + 10.47 (57.35)	341.29 + 9.33 (51.09)	351.09 + 9.41 (51.55)	365.12 + 10.38 (56.83)	383.55 + 10.51 (57.55)	0.014*
Global Efficiency	5.47 + 0.15 (0.80)	5.41 + 0.14 (0.77)	5.36 + 0.16 (0.88)	5.32 + 0.20 (1.09)	5.22 + 0.23 (1.23)	0.99
Radius	380.51 + 17.46 (95.62)	418.33 + 15.68 (85.86)	426.48 + 17.47 (95.68)	441.42 + 19.34 (105.93)	458.49 + 19.17 (104.99)	0.055

Diameter	909.17 + 29.68 (162.57)	939.53 + 25.07 (137.33)	978.45 + 24.02 (131.56)	1050.08 + 27.60 (151.16)	1125.11 + 26.34 (144.29)	< 0.001**
Assortativity	8.25 + 0.54 (2.94)	8.45 + 0.53 (2.90)	8.37 + 0.55 (3.04)	8.46 + 0.57 (3.12)	8.67 + 0.59 (3.21)	0.99
Avg. Degree Centrality	1371.76 + 44.52 (243.84)	1323.73 + 28.13 (154.07)	1246.24 + 14.78 (80.96)	1151.24 + 27.63 (151.35)	1005.13 + 41.71 (228.45)	< 0.001**
Avg. Strength	325.74 + 13.93 (76.29)	315.00 + 10.40 (56.94)	299.71 + 10.04 (54.97)	280.68 + 13.20 (72.31)	246.04 + 15.64 (85.67)	< 0.001**
Avg. Eigenvector Centrality	2.32 + 0.03 (0.17)	2.30 + 0.03 (0.17)	2.25 + 0.03 (0.16)	2.16 + 0.02 (0.13)	2.05 + 0.02 (0.12)	< 0.001**
Avg. Betweenness Centrality	5600.25 + 190.38 (1042.78)	5704.47 + 160.09 (876.86)	5747.65 + 155.90 (853.90)	5727.50 + 191.92 (1051.21)	5540.19 + 194.97 (1067.90)	0.99
Avg. Clustering Coefficient	5.77 + 0.15 (0.80)	5.75 + 0.14 (0.77)	5.74 + 0.16 (0.90)	5.66 + 0.19 (1.06)	5.38 + 0.22 (1.20)	0.72
Avg. Local Efficiency	7.01 + 0.16 (0.89)	6.98 + 0.15 (0.84)	6.90 + 0.17 (0.91)	6.72 + 0.19 (1.01)	6.32 + 0.20 (1.10)	0.055
Avg. Participation Coefficient	12.69 + 0.54 (2.96)	12.19 + 0.56 (3.06)	11.32 + 0.60 (3.31)	10.31 + 0.64 (3.48)	9.31 + 0.63 (3.43)	0.0011**

Table S5. Area-under-the-curve (AUC) values for each significant nodal measure for within-layer analysis. Data are shown as mean ± standard error (standard deviation). One-way ANOVA was used to compare layers. Node long names can be found in Table S1. P values shown are false discovery rate corrected (FDR, Bonferroni-Holm method, alpha = 0.01).
* p < 0.05, ** p < 0.01.

Node	Layer 1 (Superficial)	Layer 2	Layer 3	Layer 4	Layer 5 (Deep)	P value
Degree centrality						
Left G_and_S_cingul-Mid-Post (8)	2453.70 + 102.00 (558.67)	2369.50 + 83.28 (456.16)	2181.00 + 78.71 (431.10)	1948.07 + 92.23 (505.15)	1658.67 + 103.09 (564.65)	< 0.001**
G_cingul-Post-ventral (10)	1235.83 + 105.41 (577.37)	1119.87 + 102.18 (559.66)	802.23 + 83.68 (458.31)	505.50 + 69.92 (382.98)	281.77 + 53.47 (292.89)	< 0.001**
Left Lat_Fis-post (41)	1940.63 + 129.80 (710.95)	1876.60 + 121.41 (665.00)	1655.17 + 125.67 (688.31)	1368.37 + 137.19 (751.44)	1073.03 + 131.74 (721.57)	0.0012**
Left S_circular_insula_sup (49)	1753.07 + 113.61 (622.26)	1805.33 + 103.36 (566.13)	1701.57 + 90.66 (496.56)	1408.00 + 97.00 (531.31)	1014.70 + 105.06 (575.45)	< 0.001**
Right G_and_S_subcentral (78)	1617.93 + 104.91 (574.59)	1464.47 + 102.18 (559.67)	1301.80 + 98.82 (541.24)	1053.93 + 103.29 (565.77)	820.33 + 118.63 (649.79)	< 0.001**
Right G_and_S_cingul-Mid-Post (82)	2416.90 + 102.21 (559.83)	2355.47 + 94.21 (516.03)	2198.57 + 93.25 (510.73)	2015.23 + 100.85 (552.35)	1734.57 + 104.51 (572.42)	0.0014**

Right G_cingul-Post-ventral (84)	1289.37 + 134.01 (734.02)	1163.60 + 111.91 (612.97)	795.47 + 94.86 (519.57)	472.33 + 73.18 (400.84)	223.30 + 44.61 (244.34)	< 0.001**
Right G_oc-temp_lat-fusifor (95)	1703.73 + 137.81 (754.82)	1564.90 + 128.28 (702.64)	1361.43 + 109.30 (598.69)	1085.97 + 101.49 (555.90)	783.80 + 109.15 (597.82)	< 0.001**
Right G_temp_sup-G_T_transv (107)	1137.67 + 110.64 (605.98)	1008.40 + 111.03 (608.14)	827.27 + 93.86 (514.10)	637.50 + 87.57 (479.64)	448.97 + 88.05 (482.26)	0.0012**
Right G_temp_sup-Lateral (108)	1631.83 + 108.81 (595.98)	1409.97 + 96.23 (527.08)	1180.43 + 98.23 (538.00)	1022.60 + 109.24 (598.31)	866.70 + 115.06 (630.22)	< 0.001**
Right G_temp_sup-Plan_tempo (110)	1525.93 + 123.89 (678.59)	1423.33 + 120.28 (658.81)	1231.83 + 108.30 (593.17)	949.63 + 101.54 (556.14)	722.20 + 109.61 (600.35)	< 0.001**
Right Lat_Fis-post (115)	2042.73 + 143.53 (786.16)	2004.73 + 134.18 (734.91)	1783.97 + 116.03 (635.50)	1426.77 + 112.17 (614.40)	996.20 + 108.13 (592.24)	< 0.001**
Right S_circular_insula_inf (122)	1259.37 + 130.51 (714.81)	1198.43 + 120.04 (657.48)	931.47 + 105.74 (579.14)	646.30 + 102.02 (558.78)	505.97 + 102.45 (561.13)	< 0.001**
Right S_circular_insula_sup (123)	1481.80 + 109.33 (598.84)	1550.13 + 104.59 (572.85)	1360.07 + 88.90 (486.94)	878.80 + 100.41 (549.96)	562.57 + 101.58 (556.38)	< 0.001**
Right S_temporal_transverse (148)	1143.17 + 130.21 (713.17)	1019.17 + 127.48 (698.26)	672.10 + 90.28 (494.50)	425.37 + 82.21 (450.30)	265.77 + 74.80 (409.69)	< 0.001**
Strength						
G_cingul-Post-ventral (10)	264.18 + 25.45 (139.42)	236.06 + 24.36 (133.40)	162.14 + 19.10 (104.60)	95.76 + 14.31 (78.40)	48.61 + 9.10 (49.86)	< 0.001**
Left S_circular_insula_sup (49)	418.19 + 32.81 (179.69)	429.25 + 31.70 (173.63)	398.91 + 30.56 (167.39)	325.88 + 31.59 (173.00)	229.18 + 29.67 (162.50)	0.0040**
Right G_cingul-Post-ventral (84)	283.14 + 32.94 (180.44)	248.07 + 27.59 (151.14)	163.77 + 23.03 (126.16)	92.58 + 16.54 (90.61)	40.48 + 9.01 (49.33)	< 0.001**
Right G_oc-temp_lat-fusifor (95)	421.04 + 39.58 (216.80)	382.91 + 36.23 (198.45)	328.21 + 31.17 (170.73)	257.55 + 29.28 (160.38)	184.46 + 30.90 (169.24)	0.0010**
Right G_temp_sup-G_T_transv (107)	250.02 + 28.13 (154.05)	217.63 + 27.03 (148.04)	172.72 + 22.65 (124.04)	130.90 + 20.46 (112.07)	91.83 + 20.57 (112.68)	0.0044**
Right G_temp_sup-Plan_tempo (110)	361.66 + 33.53 (183.64)	333.42 + 31.29 (171.36)	285.03 + 28.22 (154.56)	220.65 + 28.59 (156.59)	170.30 + 31.81 (174.22)	0.0093**
Right Lat_Fis-post (115)	511.70 + 42.28 (231.60)	497.40 + 39.74 (217.66)	429.71 + 32.93 (180.37)	330.49 + 29.25 (160.23)	220.23 + 26.59 (145.65)	< 0.001**
Right S_circular_insula_sup (123)	340.46 + 30.37 (166.34)	353.82 + 27.97 (153.18)	299.25 + 22.66 (124.13)	186.79 + 25.47 (139.50)	116.80 + 25.01 (137.01)	< 0.001**
Right S_temporal_transverse (148)	247.39 + 31.99 (175.20)	217.66 + 30.16 (165.19)	135.79 + 20.74 (113.61)	81.99 + 17.37 (95.16)	49.13 + 13.78 (75.47)	< 0.001**
Eigenvector centrality						
G_cingul-Post-ventral (10)	1.84 + 0.23 (1.26)	1.73 + 0.22 (1.19)	1.24 + 0.18 (0.97)	0.76 + 0.14 (0.75)	0.41 + 0.10 (0.52)	< 0.001**

Right G_cingul-Post-ventral (84)	1.96 + 0.24 (1.33)	1.79 + 0.21 (1.14)	1.24 + 0.18 (0.99)	0.73 + 0.14 (0.75)	0.35 + 0.09 (0.49)	< 0.001**
Right S_circular_insula_sup (123)	2.57 + 0.25 (1.39)	2.78 + 0.28 (1.51)	2.43 + 0.25 (1.38)	1.57 + 0.25 (1.35)	1.03 + 0.23 (1.24)	< 0.001**
Right S_temporal_transverse (148)	1.84 + 0.27 (1.49)	1.60 + 0.26 (1.45)	0.95 + 0.19 (1.06)	0.53 + 0.16 (0.86)	0.33 + 0.15 (0.80)	< 0.001**
Betweenness centrality						
Right G_cingul-Post-ventral (84)	1809.80 + 482.95 (2645.25)	1206.27 + 343.75 (1882.77)	410.07 + 222.18 (1216.94)	138.33 + 64.88 (355.38)	12.67 + 10.27 (56.26)	0.0033**
Right G_front_inf-Opercular (86)	7844.80 + 1089.17 (5965.60)	5032.47 + 689.14 (3774.59)	3672.47 + 574.83 (3148.48)	2797.33 + 513.13 (2810.54)	2104.87 + 581.72 (3186.20)	< 0.001**
Right S_circular_insula_sup (123)	4576.87 + 797.64 (4368.84)	6260.67 + 1189.89 (6517.27)	4952.67 + 849.55 (4653.15)	1833.93 + 452.36 (2477.70)	1200.27 + 317.18 (1737.29)	0.0022**
Clustering coefficient						
G_cingul-Post-ventral (10)	5.54 + 0.30 (1.67)	5.35 + 0.32 (1.78)	4.85 + 0.37 (2.04)	4.32 + 0.38 (2.10)	3.00 + 0.43 (2.37)	0.0017**
Right G_cingul-Post-ventral (84)	5.83 + 0.31 (1.67)	5.90 + 0.29 (1.61)	5.34 + 0.34 (1.88)	4.22 + 0.38 (2.08)	2.68 + 0.39 (2.13)	< 0.001**
Right G_Ins_lg_and_S_cent_ins (91)	6.45 + 0.22 (1.20)	6.17 + 0.32 (1.74)	6.10 + 0.31 (1.72)	5.40 + 0.34 (1.85)	4.27 + 0.46 (2.51)	0.0078**
Right S_circular_insula_inf (122)	6.01 + 0.22 (1.21)	6.04 + 0.24 (1.33)	5.42 + 0.39 (2.16)	4.39 + 0.50 (2.72)	3.61 + 0.56 (3.07)	0.0063**
Right S_collat_transv_post (125)	7.74 + 0.36 (1.98)	7.53 + 0.36 (2.00)	7.17 + 0.36 (1.97)	6.43 + 0.41 (2.26)	5.20 + 0.49 (2.67)	0.0092**
Right S_temporal_transverse (148)	6.39 + 0.34 (1.88)	5.95 + 0.32 (1.78)	5.16 + 0.35 (1.91)	3.85 + 0.47 (2.55)	3.06 + 0.49 (2.67)	< 0.001**
Local efficiency						
G_cingul-Post-ventral (10)	6.56 + 0.34 (1.87)	6.26 + 0.36 (1.98)	5.49 + 0.42 (2.29)	4.69 + 0.43 (2.35)	3.16 + 0.46 (2.51)	< 0.001**
Right G_cingul-Post-ventral (84)	6.74 + 0.34 (1.88)	6.70 + 0.34 (1.85)	5.87 + 0.40 (2.17)	4.51 + 0.43 (2.34)	2.81 + 0.42 (2.32)	< 0.001**
Right G_Ins_lg_and_S_cent_ins (91)	7.11 + 0.25 (1.40)	6.85 + 0.35 (1.89)	6.70 + 0.33 (1.83)	5.91 + 0.37 (2.00)	4.67 + 0.48 (2.64)	0.0029**
Right G_temp_sup-G_T_transv (107)	7.18 + 0.31 (1.71)	6.62 + 0.34 (1.85)	5.79 + 0.42 (2.29)	5.15 + 0.48 (2.62)	4.19 + 0.55 (3.00)	0.0019**
Right S_circular_insula_inf (122)	6.88 + 0.26 (1.41)	6.89 + 0.28 (1.55)	6.14 + 0.43 (2.33)	4.97 + 0.52 (2.86)	4.03 + 0.59 (3.24)	< 0.001**
Right S_circular_insula_sup (123)	7.24 + 0.28 (1.51)	7.09 + 0.27 (1.49)	6.90 + 0.29 (1.61)	6.15 + 0.36 (1.97)	4.73 + 0.52 (2.86)	< 0.001**
Right S_collat_transv_post (125)	8.53 + 0.36 (1.95)	8.33 + 0.36 (1.99)	7.97 + 0.37 (2.05)	7.16 + 0.44 (2.43)	5.86 + 0.53 (2.91)	0.0068**
Right S_temporal_transverse (148)	7.13 + 0.34 (1.85)	6.64 + 0.33 (1.83)	5.64 + 0.36 (1.98)	4.15 + 0.48 (2.65)	3.24 + 0.51 (2.77)	< 0.001**
Participation coefficient						
G_cingul-Post-ventral (10)	14.89 + 1.12 (6.15)	13.98 + 1.04 (5.71)	11.66 + 1.12 (6.15)	8.95 + 1.10 (6.00)	4.91 + 0.93 (5.10)	< 0.001**
Left G_oc-temp_med-Parahip (23)	13.20 + 1.09 (5.99)	12.26 + 1.14 (6.26)	9.73 + 1.29 (7.06)	6.82 + 1.23 (6.75)	3.98 + 1.09 (6.00)	< 0.001**
Right G_cingul-Post-ventral (84)	14.06 + 1.30 (7.10)	13.67 + 1.16 (6.34)	11.11 + 1.27 (6.95)	7.85 + 1.25 (6.84)	4.21 + 0.92 (5.04)	< 0.001**
Right G_oc-temp_med-Parahip (97)	9.93 + 1.19 (6.52)	9.37 + 1.23 (6.73)	6.70 + 1.17 (6.41)	4.54 + 1.16 (6.35)	2.77 + 0.90 (4.95)	0.0032**

Right G_temp_sup-G_T_transv (107)	11.29 + 1.01 (5.55)	10.65 + 1.10 (6.00)	9.43 + 1.18 (6.45)	6.59 + 1.08 (5.93)	3.56 + 0.89 (4.86)	< 0.001**
Right Lat_Fis-ant-Vertical (114)	8.14 + 1.04 (5.67)	8.62 + 1.03 (5.64)	6.72 + 1.03 (5.67)	3.72 + 0.75 (4.09)	1.79 + 0.63 (3.47)	< 0.001**
Right S_circular_insula_sup (123)	14.17 + 0.80 (4.39)	14.52 + 0.92 (5.06)	12.45 + 1.07 (5.84)	8.30 + 1.24 (6.79)	5.94 + 1.09 (5.97)	< 0.001**
Right S_oc-temp_lat (134)	11.08 + 0.98 (5.38)	9.72 + 1.12 (6.15)	7.70 + 1.12 (6.11)	5.45 + 0.91 (4.99)	4.87 + 0.82 (4.51)	0.0038**
Right S_temporal_transverse (148)	10.84 + 1.21 (6.64)	10.67 + 1.18 (6.48)	8.30 + 1.14 (6.24)	5.03 + 0.98 (5.35)	3.31 + 0.80 (4.36)	< 0.001**

Table S6. Area-under-the-curve (AUC) values for each global measure for the multilayer analysis. NaN values are due to global s that cannot be derived for specific layers. Data are shown as mean ± standard error (standard deviation). One-way ANOVA was used to compare layers with an FDR correction (alpha = 0.05). Graph density and average degree centrality do not have standard error and standard deviation since they are consistent across all participants due to thresholding. Shaded rows have significant differences between layers. P values shown are false discovery rate corrected (FDR, Benjamini-Hochberg method, alpha = 0.05).
* p < 0.05, ** p < 0.01.

Measures (AUC)	Layer 1 (Superficial)	Layer 2	Layer 3	Layer 4	Layer 5 (Deep)	Multilayer	p value
Modularity	NaN	NaN	NaN	NaN	NaN	13.44 + 0.58 (3.20)	NaN
Transitivity	NaN	NaN	NaN	NaN	NaN	6.88 + 0.26 (1.42)	NaN
Largest Cluster Size	NaN	NaN	NaN	NaN	NaN	26720.50 + 198.88 (1089.31)	NaN
Graph Density	NaN	NaN	NaN	NaN	NaN	7.98	NaN
Characteristic Path Length	NaN	NaN	NaN	NaN	NaN	356.98 + 8.08 (44.27)	NaN
Global Efficiency	NaN	NaN	NaN	NaN	NaN	5.20 + 0.12 (0.68)	NaN
Radius	NaN	NaN	NaN	NaN	NaN	282.04 + 20.57 (112.65)	NaN
Diameter	NaN	NaN	NaN	NaN	NaN	1276.96 + 32.24 (176.58)	NaN
Assortativity	NaN	NaN	NaN	NaN	NaN	10.03 + 0.50 (2.74)	NaN
Avg. Degree Centrality	6120.06 + 109.88 (601.86)	6227.01 + 66.25 (362.87)	6117.00 + 29.75 (162.96)	5778.98 + 63.54 (348.03)	5243.04 + 114.25 (625.76)	5897.22	< 0.001**
Avg. Strength	1471.07 + 41.23 (225.80)	1519.12 + 38.35 (210.05)	1506.81 + 41.33 (226.37)	1425.36 + 46.45 (254.42)	1279.02 + 52.03 (285.00)	1440.27 + 36.56 (200.23)	0.0019**
Avg. Eigenvector Centrality	0.99 + 0.03 (0.18)	1.03 + 0.02 (0.13)	1.03 + 0.02 (0.08)	0.99 + 0.01 (0.08)	0.89 + 0.02 (0.13)	0.99 + 0.01 (0.06)	< 0.001**
Avg. Betweenness	61782.76 +	41292.92 +	31429.48 +	23221.57 +	16319.54 +	34809.25 +	< 0.001**

Centrality	3026.44 (16576.48)	1015.60 (5562.69)	1187.24 (6502.81)	1255.80 (6878.27)	1597.85 (8751.78)	634.48 (3475.21)	
Avg. Clustering Coefficient	6.92 + 0.22 (1.19)	6.94 + 0.21 (1.14)	7.05 + 0.20 (1.11)	7.26 + 0.20 (1.11)	7.67 + 0.21 (1.16)	7.17 + 0.21 (1.13)	0.080
Avg. Local Efficiency	8.39 + 0.21 (1.14)	8.47 + 0.21 (1.14)	8.53 + 0.21 (1.13)	8.58 + 0.21 (1.13)	8.74 + 0.21 (1.15)	8.54 + 0.21 (1.13)	0.81
Avg. Participation Coefficient	13.12 + 0.61 (3.33)	12.83 + 0.61 (3.35)	12.25 + 0.61 (3.34)	11.45 + 0.60 (3.28)	10.45 + 0.57 (3.13)	12.02 + 0.59 (3.22)	0.019*

Table S7. Area-under-the-curve (AUC) values for each significant nodal measure for multilayer analysis. Data are shown as mean ± standard error (standard deviation). One-way ANOVA was used to compare layers. Node long names can be found in Table S1. P values shown are false discovery rate corrected (FDR, Bonferroni-Holm method, alpha = 0.01).
* p < 0.05, ** p < 0.01.

Node	Layer 1 (Superficial)	Layer 2	Layer 3	Layer 4	Layer 5 (Deep)	P value
Degree centrality						
Left G_cingul-Post-ventral (10)	5076.87 + 431.15 (2361.53)	4969.63 + 452.10 (2476.26)	3985.50 + 405.98 (2223.62)	2812.13 + 373.43 (2045.38)	1630.10 + 287.36 (1573.94)	< 0.001**
Left S_circular_insula_sup (49)	7193.63 + 486.46 (2664.46)	8094.03 + 482.03 (2640.19)	8278.37 + 439.28 (2406.03)	7029.77 + 462.79 (2534.79)	5169.37 + 485.06 (2656.79)	0.0062**
Right G_cingul-Post-ventral (84)	5147.20 + 540.39 (2959.82)	5051.87 + 480.47 (2631.63)	3938.47 + 445.94 (2442.51)	2751.13 + 394.04 (2158.26)	1539.73 + 280.11 (1534.20)	< 0.001**
Right G_temp_sup-G_T_transv (107)	5177.73 + 519.32 (2844.45)	4754.93 + 518.62 (2840.60)	4123.13 + 457.67 (2506.78)	3238.60 + 420.49 (2303.14)	2119.83 + 366.05 (2004.92)	0.0043**
Right S_circular_insula_sup (123)	5919.10 + 492.79 (2699.12)	6862.40 + 475.47 (2604.27)	6705.53 + 425.99 (2333.27)	4324.00 + 461.73 (2528.98)	2521.00 + 415.35 (2274.98)	< 0.001**
Right S_temporal_transverse (148)	4721.87 + 572.15 (3133.78)	4574.57 + 570.52 (3124.88)	3376.47 + 433.26 (2373.07)	2286.10 + 407.43 (2231.56)	1349.23 + 317.51 (1739.06)	< 0.001**
Strength						
Left G_cingul-Post-ventral (10)	1076.90 + 99.01 (542.29)	1057.98 + 106.84 (585.16)	826.63 + 93.02 (509.48)	557.68 + 78.19 (428.29)	298.81 + 52.85 (289.47)	< 0.001**
Right G_cingul-Post-ventral (84)	1122.08 + 128.86 (705.81)	1087.16 + 117.50 (643.58)	830.00 + 107.62 (589.48)	564.44 + 90.62 (496.36)	302.89 + 62.34 (341.47)	< 0.001**
Right G_temp_sup-G_T_transv (107)	1137.14 + 124.93 (684.29)	1043.00 + 123.51 (676.49)	889.30 + 111.09 (608.45)	684.54 + 96.70 (529.64)	431.61 + 81.54 (446.62)	0.0068**

Right S_circular_insula_sup (123)	1322.47 + 126.86 (694.86)	1550.80 + 120.67 (660.95)	1498.64 + 105.59 (578.36)	938.46 + 115.63 (633.34)	529.45 + 101.39 (555.31)	< 0.001**
Right S_temporal_transverse (148)	1003.81 + 132.47 (725.58)	981.60 + 131.61 (720.83)	704.36 + 99.97 (547.55)	458.87 + 84.51 (462.87)	250.96 + 55.15 (302.06)	< 0.001**
Eigenvector centrality						
Left G_cingul-Post-ventral (10)	0.72 + 0.09 (0.47)	0.71 + 0.09 (0.48)	0.55 + 0.08 (0.42)	0.38 + 0.07 (0.36)	0.21 + 0.05 (0.25)	< 0.001**
Right G_cingul-Post-ventral (84)	0.79 + 0.11 (0.59)	0.75 + 0.10 (0.52)	0.56 + 0.08 (0.45)	0.38 + 0.07 (0.37)	0.20 + 0.04 (0.24)	< 0.001**
Right S_circular_insula_sup (123)	0.95 + 0.12 (0.66)	1.13 + 0.12 (0.68)	1.09 + 0.11 (0.62)	0.67 + 0.10 (0.56)	0.37 + 0.08 (0.45)	< 0.001**
Betweenness centrality						
Left G_and_S_cingul-Mid-Post (8)	105111.27 + 21687.37 (118786.64)	88184.53 + 13701.49 (75046.13)	53839.20 + 10915.52 (59786.76)	25821.60 + 6124.16 (33543.42)	11866.60 + 3565.61 (19529.64)	< 0.001**
Left G_cingul-Post-dorsal (9)	52676.60 + 12036.96 (65929.12)	33667.53 + 9945.04 (54471.23)	8431.53 + 2396.37 (13125.47)	6178.80 + 1168.20 (6398.47)	4070.73 + 3076.98 (16853.32)	< 0.001**
Left G_cingul-Post-ventral (10)	41191.27 + 8343.09 (45696.99)	34999.13 + 7045.00 (38587.07)	19735.00 + 3091.28 (16931.64)	16520.60 + 2274.77 (12459.45)	118.60 + 99.52 (545.10)	< 0.001**
Left G_front_inf-Opercular (12)	92545.73 + 13077.13 (71626.40)	33567.13 + 7110.37 (38945.10)	19672.27 + 4132.23 (22633.16)	11064.33 + 3243.25 (17763.99)	3848.73 + 1381.32 (7565.78)	< 0.001**
Left G_front_inf-Orbital (13)	49827.53 + 8908.29 (48792.71)	16566.47 + 3174.81 (17389.12)	9858.60 + 1910.07 (10461.87)	9051.00 + 1659.84 (9091.32)	4139.80 + 2511.86 (13758.02)	< 0.001**
Left G_front_middle (15)	197365.53 + 31853.65 (174469.61)	95720.40 + 13721.65 (75156.58)	75488.80 + 11051.19 (60529.87)	66872.73 + 11962.40 (65520.76)	38275.20 + 6217.33 (34053.73)	< 0.001**
Left G_front_sup (16)	313885.67 + 40031.09 (219259.30)	142010.27 + 18217.86 (99783.30)	80349.13 + 12807.12 (70147.47)	65373.80 + 8248.62 (45179.57)	88886.93 + 15911.67 (87151.82)	< 0.001**
Left G_insular_short (18)	31900.33 + 6397.00 (35037.80)	17459.27 + 3608.71 (19765.71)	9014.27 + 1829.48 (10020.46)	8794.20 + 1569.99 (8599.19)	2107.13 + 1122.81 (6149.87)	< 0.001**
Left G_occipital_middle (19)	237544.73 + 48014.28 (262985.06)	140370.93 + 33163.87 (181645.97)	104380.53 + 16827.03 (92165.41)	82597.40 + 15025.27 (82296.81)	41015.93 + 11499.84 (62987.20)	0.0043**
Left G_oc-temp_lat-fusifor (21)	96157.40 + 19979.53	39225.13 + 15223.08 (83380.22)	20072.27 + 4128.17 (22610.92)	14707.20 + 4454.70 (24399.39)	4135.80 + 2410.01 (13200.16)	< 0.001**

	(109432.37)					
Left G_oc-temp_med-Lingual (22)	69466.07 + 12899.34 (70652.59)	31268.93 + 7764.06 (42525.49)	29618.33 + 7506.47 (41114.63)	17770.00 + 4370.58 (23938.66)	13969.33 + 5456.86 (29888.45)	0.0023**
Left G_oc-temp_med-Parahip (23)	41862.33 + 9796.64 (53658.40)	41384.53 + 8338.12 (45669.78)	28466.33 + 4438.51 (24310.71)	19811.60 + 3127.25 (17128.67)	1506.27 + 982.02 (5378.73)	0.0025**
Left G_pariet_inf-Angular (25)	98068.00 + 22425.47 (122829.36)	27804.47 + 4884.88 (26755.60)	16650.27 + 3040.58 (16653.94)	19055.67 + 4087.76 (22389.59)	43204.93 + 10614.68 (58138.97)	< 0.001**
Left G_postcentral (28)	116097.13 + 27145.36 (148681.25)	35613.33 + 9633.32 (52763.85)	24237.13 + 7107.45 (38929.11)	25213.00 + 7376.22 (40401.23)	41871.00 + 11103.41 (60815.87)	0.0035**
Left G_precentral (29)	170934.87 + 26909.80 (147391.07)	59417.27 + 11538.89 (63201.10)	35258.87 + 5729.28 (31380.55)	32005.93 + 5076.26 (27803.84)	24004.80 + 6956.28 (38101.14)	< 0.001**
Left G_rectus (31)	57752.80 + 11331.03 (62062.61)	44832.00 + 9037.83 (49502.24)	28576.60 + 7376.67 (40403.71)	15513.20 + 6767.90 (37069.33)	5153.53 + 1704.78 (9337.49)	0.0024**
Left G_temp_sup-G_T_transv (33)	51038.60 + 9510.61 (52091.75)	19368.47 + 2719.68 (14896.29)	17839.13 + 3444.43 (18865.91)	9286.87 + 1348.75 (7387.42)	331.33 + 136.49 (747.59)	< 0.001**
Left G_temp_sup-Lateral (34)	83950.93 + 11879.40 (65066.14)	39945.93 + 7011.60 (38404.09)	41298.47 + 16601.94 (90932.58)	12945.47 + 2762.52 (15130.93)	23871.73 + 6904.94 (37819.94)	0.0034**
Left G_temporal_middle (38)	187415.87 + 28244.91 (154703.74)	82059.53 + 17024.94 (93249.45)	43896.00 + 7876.16 (43139.48)	31837.60 + 6662.65 (36492.85)	24252.00 + 6890.19 (37739.15)	< 0.001**
Left S_circular_insula_ant (47)	24766.73 + 5261.14 (28816.47)	36282.00 + 5509.23 (30175.28)	25445.67 + 4485.30 (24567.01)	13956.87 + 3600.78 (19722.27)	3111.53 + 1396.26 (7647.64)	< 0.001**
Left S_circular_insula_sup (49)	48917.93 + 12875.21 (70520.42)	106401.80 + 16440.63 (90049.06)	92955.93 + 16929.26 (92725.38)	35489.13 + 6671.83 (36543.14)	6466.47 + 3290.52 (18022.93)	< 0.001**
Left S_front_inf (52)	62831.73 + 10360.31 (56745.74)	59448.40 + 6516.83 (35694.13)	48399.93 + 6938.10 (38001.54)	30227.87 + 4508.65 (24694.92)	16950.27 + 4099.20 (22452.26)	< 0.001**
Left S_orbital_med-olfact (63)	596.67 + 421.62 (2309.28)	14329.20 + 1963.18 (10752.78)	18730.47 + 3237.31 (17731.47)	13094.47 + 2811.08 (15396.94)	2033.27 + 1853.08 (10149.73)	< 0.001**
Left S_temporal_transverse (74)	14236.27 + 3659.12 (20041.80)	22790.07 + 4035.57 (22103.73)	20026.27 + 3043.27 (16668.68)	12175.60 + 2697.16 (14772.95)	2630.53 + 1356.82 (7431.60)	0.0084**
Right G_and_S_cingul-Ant (80)	64216.40 + 11745.09 (64330.52)	67585.53 + 9000.28 (49296.54)	41575.13 + 7966.16 (43632.48)	16670.13 + 3050.32 (16707.30)	11471.93 + 2845.97 (15588.01)	< 0.001**

Right G_and_S_cingul-Mid-Ant (81)	78713.80 + 10416.55 (57053.79)	64613.47 + 10780.29 (59046.05)	31945.73 + 6193.80 (33924.82)	21900.40 + 3748.59 (20531.87)	10724.93 + 3149.80 (17252.16)	< 0.001**
Right G_and_S_cingul-Mid-Post (82)	126618.73 + 28915.78 (158378.24)	93623.27 + 16875.42 (92430.48)	57820.60 + 13101.10 (71757.68)	27995.00 + 7217.24 (39530.48)	15588.13 + 3819.77 (20921.73)	0.0011**
Right G_cingul-Post-dorsal (83)	35260.33 + 5941.80 (32544.58)	16464.00 + 3574.12 (19576.29)	12573.53 + 3781.53 (20712.29)	7328.67 + 2412.00 (13211.09)	3385.47 + 2361.34 (12933.58)	< 0.001**
Right G_cingul-Post-ventral (84)	38386.87 + 9970.24 (54609.28)	28352.87 + 5042.84 (27620.76)	19435.40 + 2500.57 (13696.21)	13525.60 + 1753.23 (9602.85)	7.33 + 5.36 (29.34)	< 0.001**
Right G_front_inf-Opercular (86)	152062.27 + 19806.48 (108484.57)	28949.33 + 4864.10 (26641.77)	20385.87 + 4250.10 (23278.77)	10186.73 + 2443.80 (13385.25)	3269.87 + 1214.77 (6653.59)	< 0.001**
Right G_front_inf-Orbital (87)	43334.73 + 8432.02 (46184.09)	23913.67 + 3371.55 (18466.74)	9534.67 + 1398.99 (7662.56)	7453.20 + 1589.05 (8703.59)	937.53 + 373.98 (2048.37)	< 0.001**
Right G_front_inf-Triangul (88)	52534.00 + 7659.68 (41953.79)	23017.73 + 5431.49 (29749.51)	21416.40 + 5105.64 (27964.73)	11666.53 + 1624.36 (8896.98)	8153.33 + 2783.86 (15247.80)	< 0.001**
Right G_front_middle (89)	214042.27 + 20934.55 (114663.24)	98732.93 + 13682.04 (74939.64)	69602.60 + 10903.46 (59720.70)	50030.07 + 6732.17 (36873.60)	27503.33 + 4564.23 (24999.32)	< 0.001**
Right G_front_sup (90)	345289.40 + 51019.32 (279444.30)	148996.13 + 32052.34 (175557.88)	93208.67 + 14036.72 (76882.28)	66308.47 + 10686.41 (58531.86)	81284.80 + 23421.57 (128285.23)	< 0.001**
Right G_insular_short (92)	37965.13 + 10619.85 (58167.32)	14086.80 + 2894.45 (15853.55)	9767.13 + 1585.03 (8681.58)	6942.67 + 1383.97 (7580.31)	811.33 + 503.80 (2759.43)	< 0.001**
Right G_occipital_middle (93)	159380.87 + 28486.50 (156026.98)	76258.13 + 8756.12 (47959.26)	53203.20 + 9637.67 (52787.72)	37151.67 + 7178.59 (39318.77)	19012.33 + 4927.31 (26988.00)	< 0.001**
Right G_oc-temp_lat-fusifor (95)	110338.87 + 25381.37 (139019.46)	52452.33 + 12697.63 (69547.78)	33206.60 + 10774.91 (59016.59)	13479.07 + 2749.87 (15061.67)	1109.27 + 352.26 (1929.42)	< 0.001**
Right G_pariet_inf-Angular (99)	120177.87 + 24578.46 (134621.77)	50247.67 + 11493.28 (62951.31)	26490.67 + 4538.14 (24856.40)	37551.47 + 11515.87 (63075.04)	30817.40 + 10703.71 (58626.65)	0.0019**
Right G_pariet_inf-Supramar (100)	109984.67 + 18074.86 (99000.11)	45252.60 + 8881.63 (48646.71)	27305.13 + 5030.82 (27554.96)	23405.27 + 4688.14 (25678.02)	14616.80 + 4831.58 (26463.64)	< 0.001**
Right G_postcentral (102)	84326.40 + 22112.45 (121114.87)	17218.27 + 3998.58 (21901.14)	15242.80 + 3379.61 (18510.90)	15186.33 + 3873.90 (21218.24)	13728.80 + 3577.11 (19592.66)	< 0.001**

Right G_precentral (103)	190926.87 + 37351.28 (204581.40)	66875.33 + 15460.99 (84683.34)	45879.67 + 12249.57 (67093.64)	51201.80 + 12121.71 (66393.36)	34691.80 + 8540.63 (46778.94)	< 0.001**
Right G_temp_sup-G_T_transv (107)	36130.53 + 6608.81 (36197.94)	16648.60 + 2333.59 (12781.57)	16394.67 + 2539.55 (13909.67)	14561.47 + 2598.72 (14233.77)	765.13 + 424.82 (2326.84)	< 0.001**
Right G_temp_sup-Lateral (108)	121087.73 + 17298.97 (94750.34)	35942.07 + 6505.30 (35630.99)	25542.07 + 6751.03 (36976.92)	10383.07 + 2624.35 (14374.16)	4302.53 + 1699.32 (9307.54)	< 0.001**
Right G_temp_sup-Plan_polar (109)	24289.13 + 6501.03 (35607.61)	42593.20 + 8815.28 (48283.29)	40997.87 + 7500.53 (41082.10)	22264.40 + 4818.90 (26394.22)	1445.00 + 777.33 (4257.63)	0.0035**
Right G_temp_sup-Plan_tempo (110)	42973.00 + 7071.08 (38729.90)	23677.33 + 3275.76 (17942.08)	15663.87 + 2486.45 (13618.85)	8484.20 + 1639.26 (8978.59)	2561.20 + 790.48 (4329.65)	< 0.001**
Right G_temporal_inf (111)	79664.73 + 11389.51 (62382.92)	28290.27 + 4688.69 (25680.99)	18152.93 + 2752.38 (15075.39)	11442.00 + 2032.76 (11133.87)	10119.40 + 3380.23 (18514.27)	< 0.001**
Right G_temporal_middle (112)	173375.93 + 26035.41 (142601.79)	54950.33 + 9840.76 (53900.07)	33286.60 + 7269.20 (39815.04)	25858.80 + 6018.67 (32965.62)	9680.13 + 3832.14 (20989.49)	< 0.001**
Right Lat_Fis-ant-Horizont (113)	18865.07 + 4435.12 (24292.13)	33603.73 + 4600.21 (25196.39)	25345.60 + 3046.15 (16684.45)	16119.67 + 2265.73 (12409.91)	1435.87 + 582.78 (3192.02)	< 0.001**
Right Lat_Fis-ant-Vertical (114)	15767.00 + 4736.48 (25942.75)	24265.33 + 4096.57 (22437.84)	29988.27 + 3615.07 (19800.57)	16808.27 + 2649.16 (14510.03)	684.27 + 404.03 (2212.94)	< 0.001**
Right S_cingul-Marginalis (120)	52174.07 + 9268.89 (50767.80)	72520.80 + 13369.77 (73229.26)	54375.73 + 9053.30 (49586.99)	28907.67 + 5690.32 (31167.17)	12463.80 + 5053.25 (27677.81)	0.0043**
Right S_circular_insula_ant (121)	19154.87 + 4957.66 (27154.23)	30905.27 + 5141.69 (28162.20)	21499.13 + 3704.73 (20291.63)	11061.87 + 1578.91 (8648.05)	2136.67 + 824.68 (4516.98)	< 0.001**
Right S_circular_insula_inf (122)	14624.40 + 3654.62 (20017.19)	29294.47 + 4666.89 (25561.62)	40747.33 + 6191.92 (33914.53)	23195.40 + 4683.69 (25653.62)	7728.40 + 3794.70 (20784.43)	0.0017**
Right S_circular_insula_sup (123)	46867.93 + 16203.43 (88749.86)	79768.47 + 19275.33 (105575.31)	51013.67 + 5742.32 (31451.99)	18050.33 + 2963.28 (16230.55)	661.13 + 332.72 (1822.38)	0.0035**
Right S_front_inf (126)	107645.47 + 17722.77 (97071.63)	74946.60 + 11637.13 (63739.16)	45043.93 + 7252.59 (39724.07)	27807.07 + 4862.20 (26631.35)	26214.60 + 5548.54 (30390.60)	< 0.001**
Right S_front_sup (128)	79632.47 + 13830.82 (75754.54)	36168.53 + 7678.46 (42056.68)	29531.80 + 5614.97 (30754.47)	26537.53 + 4818.88 (26394.08)	29881.60 + 9005.54 (49325.38)	0.0083**
Right S_precentral-inf-part (142)	57704.07 + 9423.79 (51616.23)	30839.20 + 5082.89 (27840.12)	17550.73 + 4074.14 (22315.01)	14715.93 + 2876.74 (15756.57)	11496.40 + 2844.94 (15582.39)	< 0.001**
Right S_temporal_sup (147)	174853.93 + 26896.66	118394.60 + 18811.82	75028.93 + 18497.30	49068.60 + 8470.57 (46395.23)	66687.87 + 18400.09	0.0035**

	(147319.07)	(103036.58)	(101313.89)		(100781.46)	
Right S_temporal_transverse (148)	30180.20 + 6426.10 (35197.21)	34518.00 + 7055.62 (38645.19)	26316.60 + 4549.83 (24920.47)	15185.87 + 2159.35 (11827.24)	669.53 + 412.91 (2261.62)	< 0.001**
Clustering coefficient						
Left G_oc-temp_lat-fusifor (21)	6.92 + 0.29 (1.57)	7.23 + 0.28 (1.53)	7.52 + 0.26 (1.40)	7.96 + 0.24 (1.33)	8.71 + 0.26 (1.45)	0.0064**
Right G_front_inf-Opercular (86)	5.84 + 0.30 (1.66)	6.51 + 0.30 (1.64)	7.11 + 0.31 (1.67)	7.80 + 0.34 (1.88)	8.67 + 0.42 (2.29)	< 0.001**
Right G_front_inf-Orbital (87)	6.35 + 0.29 (1.56)	6.70 + 0.25 (1.38)	7.40 + 0.29 (1.56)	7.84 + 0.36 (1.94)	8.56 + 0.39 (2.12)	0.0016**
Right G_oc-temp_lat-fusifor (95)	6.62 + 0.22 (1.23)	6.77 + 0.21 (1.13)	6.98 + 0.20 (1.10)	7.45 + 0.20 (1.07)	8.33 + 0.24 (1.29)	< 0.001**
Right G_temp_sup-Lateral (108)	6.26 + 0.30 (1.63)	6.69 + 0.30 (1.66)	7.20 + 0.32 (1.75)	7.64 + 0.31 (1.70)	8.28 + 0.31 (1.69)	0.0096**
Right G_temp_sup-Plan_tempo (110)	6.82 + 0.24 (1.29)	6.90 + 0.24 (1.34)	7.21 + 0.26 (1.44)	7.83 + 0.28 (1.51)	8.67 + 0.32 (1.76)	< 0.001**
Right Lat_Fis-post (115)	6.30 + 0.22 (1.21)	6.09 + 0.20 (1.08)	6.20 + 0.20 (1.07)	6.78 + 0.23 (1.25)	7.63 + 0.30 (1.63)	0.0025**
Participation coefficient						
Left G_cingul-Post-ventral (10)	15.53 + 1.03 (5.66)	15.15 + 0.99 (5.44)	13.13 + 1.06 (5.80)	10.37 + 1.14 (6.24)	7.41 + 1.12 (6.16)	< 0.001**
Left G_oc-temp_med-Parahip (23)	14.71 + 1.22 (6.69)	13.78 + 1.17 (6.43)	11.82 + 1.29 (7.08)	8.88 + 1.20 (6.58)	5.26 + 0.97 (5.33)	< 0.001**
Left S_circular_insula_sup (49)	16.00 + 1.02 (5.56)	16.91 + 0.95 (5.18)	16.50 + 0.95 (5.23)	13.75 + 1.09 (5.95)	10.60 + 1.10 (6.00)	0.0094**
Right G_cingul-Post-ventral (84)	14.61 + 1.28 (7.04)	14.62 + 1.19 (6.52)	13.14 + 1.22 (6.68)	10.81 + 1.20 (6.56)	7.28 + 1.07 (5.83)	0.0072**
Right G_oc-temp_med-Parahip (97)	11.51 + 1.21 (6.65)	10.33 + 1.26 (6.91)	8.30 + 1.15 (6.32)	6.83 + 1.15 (6.30)	3.91 + 0.95 (5.18)	0.0074**
Right Lat_Fis-ant-Vertical (114)	9.76 + 1.04 (5.67)	9.88 + 1.10 (6.05)	8.38 + 1.07 (5.89)	5.57 + 0.98 (5.34)	3.62 + 0.90 (4.91)	0.0032**
Right S_circular_insula_sup (123)	15.04 + 0.95 (5.21)	15.28 + 1.03 (5.66)	13.92 + 1.15 (6.31)	10.59 + 1.23 (6.74)	7.60 + 1.15 (6.31)	< 0.001**

Table S8. Area-under-the-curve (AUC) values for each global measure for the between-layer analysis. NaN values are due to global s that cannot be derived for specific layers. Data are shown as mean ± standard error (standard deviation). One-way ANOVA was used to compare layers with an FDR correction (alpha = 0.05). Graph density and average degree centrality do not have standard error and standard deviation since they are consistent across all participants due to thresholding. Shaded rows have significant differences between layers. P values shown are false discovery rate corrected (FDR, Benjamini-Hochberg method, alpha = 0.05).

* $p < 0.05$, ** $p < 0.01$.

Measures (AUC)	Layer 1 (Superficial)	Layer 2	Layer 3	Layer 4	Layer 5 (Deep)	Multilayer	p value
Modularity	NaN	NaN	NaN	NaN	NaN	13.74 + 0.57 (3.14)	NaN
Transitivity	NaN	NaN	NaN	NaN	NaN	5.16 + 0.19 (1.03)	NaN
Largest Cluster Size	NaN	NaN	NaN	NaN	NaN	26641.40 + 202.95 (1111.58)	NaN
Graph Density	NaN	NaN	NaN	NaN	NaN	6.33 + 0.01 (0.05)	NaN
Characteristic Path Length	NaN	NaN	NaN	NaN	NaN	363.49 + 8.04 (44.03)	NaN
Global Efficiency	NaN	NaN	NaN	NaN	NaN	5.06 + 0.12 (0.64)	NaN
Radius	NaN	NaN	NaN	NaN	NaN	277.38 + 20.37 (111.56)	NaN
Diameter	NaN	NaN	NaN	NaN	NaN	1283.42 + 31.67 (173.45)	NaN
Assortativity	NaN	NaN	NaN	NaN	NaN	10.34 + 0.49 (2.69)	NaN
Avg. Degree Centrality	4748.31 + 69.28 (379.49)	4903.28 + 38.52 (210.96)	4870.76 + 15.08 (82.60)	4627.74 + 37.58 (205.85)	4237.92 + 73.16 (400.72)	4677.60 + 6.68 (36.56)	< 0.001**
Avg. Strength	1145.33 + 29.00 (158.84)	1204.12 + 28.97 (158.68)	1207.09 + 31.67 (173.45)	1144.67 + 33.91 (185.71)	1032.98 + 37.04 (202.88)	1146.84 + 28.45 (155.81)	0.0021**
Avg. Eigenvector Centrality	0.98 + 0.03 (0.14)	1.02 + 0.02 (0.11)	1.03 + 0.01 (0.08)	0.99 + 0.01 (0.06)	0.91 + 0.02 (0.09)	0.98 + 0.01 (0.06)	< 0.001**

Avg. Betweenness Centrality	46777.98 + 1942.31 (10638.44)	54155.59 + 1315.20 (7203.67)	39616.03 + 1012.14 (5543.72)	27019.47 + 1407.76 (7710.59)	14833.07 + 1259.23 (6897.06)	36480.43 + 572.22 (3134.15)	< 0.001**
Avg. Clustering Coefficient	5.53 + 0.20 (1.07)	5.37 + 0.18 (0.97)	5.46 + 0.16 (0.89)	5.73 + 0.16 (0.87)	6.28 + 0.17 (0.94)	5.67 + 0.17 (0.92)	0.0033**
Avg. Local Efficiency	7.78 + 0.20 (1.07)	7.74 + 0.19 (1.04)	7.85 + 0.19 (1.02)	7.94 + 0.18 (1.00)	8.18 + 0.19 (1.04)	7.90 + 0.19 (1.02)	0.50
Avg. Participation Coefficient	12.89 + 0.61 (3.35)	12.85 + 0.62 (3.37)	12.38 + 0.61 (3.34)	11.58 + 0.59 (3.24)	10.51 + 0.56 (3.06)	12.04 + 0.59 (3.21)	0.032*

Table S9. Area-under-the-curve (AUC) values for each significant nodal measure for between-layer analysis. Data are shown as mean ± standard error (standard deviation). One-way ANOVA was used to compare layers. Node long names can be found in Table S1. P values shown are false discovery rate corrected (FDR, Bonferroni-Holm method, alpha = 0.01).
* p < 0.05, ** p < 0.01.

Node	Layer 1 (Superficial)	Layer 2	Layer 3	Layer 4	Layer 5 (Deep)	P value
Degree centrality						
Left G_cingul-Post-ventral (10)	3841.03 + 329.56 (1805.05)	3849.77 + 350.81 (1921.47)	3183.27 + 322.75 (1767.77)	2306.63 + 304.62 (1668.47)	1348.33 + 237.56 (1301.17)	< 0.001**
Right G_cingul-Post-ventral (84)	3857.83 + 409.31 (2241.89)	3888.27 + 369.22 (2022.28)	3143.00 + 352.19 (1929.02)	2278.80 + 322.49 (1766.33)	1316.43 + 238.51 (1306.39)	< 0.001**
Right G_temp_sup-G_T_transv (107)	4040.07 + 417.16 (2284.87)	3746.53 + 410.20 (2246.77)	3295.87 + 363.96 (1993.52)	2601.10 + 334.77 (1833.60)	1670.87 + 280.79 (1537.97)	0.0068**
Right S_circular_insula_sup (123)	4437.30 + 392.73 (2151.06)	5312.27 + 374.22 (2049.67)	5345.47 + 338.21 (1852.44)	3445.20 + 363.29 (1989.82)	1958.43 + 316.42 (1733.13)	< 0.001**
Right S_temporal_transverse (148)	3578.70 + 447.70 (2452.15)	3555.40 + 445.31 (2439.09)	2704.37 + 343.23 (1879.94)	1860.73 + 326.43 (1787.91)	1083.47 + 246.27 (1348.90)	< 0.001**
			Strength			
Left G_cingul-Post-ventral (10)	812.72 + 74.37 (407.33)	821.92 + 82.64 (452.65)	664.49 + 74.04 (405.52)	461.91 + 64.09 (351.02)	250.19 + 44.38 (243.06)	< 0.001**
Right G_cingul-Post-ventral (84)	838.94 + 96.61 (529.15)	839.09 + 90.04 (493.18)	666.23 + 84.90 (464.99)	471.86 + 74.48 (407.95)	262.40 + 53.86 (295.02)	< 0.001**
Right G_temp_sup-G_T_transv (107)	887.12 + 98.99 (542.19)	825.37 + 97.21 (532.42)	716.58 + 88.47 (484.59)	553.64 + 76.77 (420.48)	339.79 + 61.55 (337.10)	0.0086**
Right S_circular_insula_sup	982.01 + 98.63 (540.19)	1196.99 + 93.44 (511.79)	1199.38 + 83.28 (456.17)	751.67 + 90.64 (496.48)	412.65 + 76.81 (420.68)	< 0.001**

(123)						
Right S_temporal_transverse (148)	756.41 + 101.77 (557.41)	763.94 + 101.97 (558.52)	568.57 + 79.29 (434.27)	376.88 + 67.49 (369.67)	201.84 + 42.29 (231.66)	< 0.001**
Eigenvector centrality						
Left G_cingul-Post-ventral (10)	0.69 + 0.08 (0.46)	0.69 + 0.09 (0.47)	0.55 + 0.08 (0.42)	0.38 + 0.07 (0.37)	0.22 + 0.05 (0.26)	0.0018**
Right G_cingul-Post-ventral (84)	0.75 + 0.10 (0.56)	0.72 + 0.09 (0.50)	0.56 + 0.08 (0.44)	0.39 + 0.07 (0.38)	0.21 + 0.05 (0.27)	0.0015**
Right S_circular_insula_sup (123)	0.89 + 0.12 (0.63)	1.08 + 0.12 (0.66)	1.08 + 0.11 (0.61)	0.67 + 0.10 (0.55)	0.36 + 0.08 (0.42)	< 0.001**
Betweenness centrality						
Left G_and_S_cingul-Mid-Post (8)	75702.67 + 15316.89 (83894.08)	116668.93 + 17455.29 (95606.56)	64943.20 + 11393.84 (62406.64)	30863.40 + 6692.49 (36656.26)	12208.07 + 3419.26 (18728.07)	< 0.001**
Left G_cingul-Post-dorsal (9)	38577.27 + 9521.32 (52150.44)	43946.80 + 10765.32 (58964.11)	14194.20 + 3648.27 (19982.39)	8228.47 + 1894.50 (10376.59)	3158.20 + 2251.49 (12331.90)	0.0018**
Left G_cingul-Post-ventral (10)	32904.73 + 6443.48 (35292.41)	38474.00 + 6997.18 (38325.12)	20040.73 + 3049.83 (16704.58)	15662.33 + 2105.77 (11533.80)	143.00 + 118.72 (650.24)	< 0.001**
Left G_front_inf-Opercular (12)	73589.33 + 11669.80 (63918.15)	43739.53 + 7197.94 (39424.74)	25929.73 + 4865.49 (26649.37)	12373.00 + 2701.49 (14796.70)	3848.67 + 1458.55 (7988.83)	< 0.001**
Left G_front_inf-Orbital (13)	39247.60 + 7283.20 (39891.76)	23116.00 + 3341.05 (18299.68)	11865.87 + 1862.56 (10201.66)	9916.87 + 1461.56 (8005.31)	4184.53 + 2431.42 (13317.41)	< 0.001**
Left G_front_middle (15)	138701.53 + 23136.59 (126724.33)	139929.07 + 15424.83 (84485.27)	103788.73 + 12776.50 (69979.75)	77956.67 + 11602.30 (63548.42)	34907.53 + 5922.10 (32436.69)	< 0.001**
Left G_front_sup (16)	225676.67 + 29970.60 (164155.76)	213360.13 + 22275.09 (122005.67)	118380.20 + 14580.33 (79859.74)	83235.80 + 9088.32 (49778.78)	76423.13 + 14209.82 (77830.39)	< 0.001**
Left G_insular_short (18)	29407.80 + 6153.58 (33704.53)	18947.80 + 3552.32 (19456.83)	9035.47 + 1675.70 (9178.17)	9099.00 + 1548.31 (8480.43)	1930.47 + 1058.46 (5797.44)	< 0.001**
Left G_oc-temp_lat-fusifor (21)	68827.13 + 13646.22 (74743.40)	55690.80 + 14512.29 (79487.06)	28297.33 + 5224.97 (28618.34)	15889.47 + 4201.53 (23012.74)	4454.60 + 1810.77 (9918.00)	< 0.001**
Left G_oc-temp_med-Parahip (23)	37669.47 + 8039.60 (44034.70)	41599.93 + 7574.38 (41486.57)	29273.73 + 4361.26 (23887.61)	20158.07 + 2951.38 (16165.35)	1459.40 + 1005.31 (5506.32)	< 0.001**
Left G_precentral (29)	125074.00 + 20356.32 (111496.18)	87534.07 + 14575.36 (79832.53)	51056.73 + 6922.60 (37916.65)	41199.53 + 6284.88 (34423.68)	20960.47 + 5480.23 (30016.44)	< 0.001**
Left G_precuneus (30)	121827.80 + 21297.97 (116653.78)	148253.67 + 14325.09 (78461.74)	155588.07 + 19908.51 (109043.41)	115986.67 + 17019.13 (93217.61)	45811.13 + 10390.61	0.0072**

					(56911.72)	
Left G_rectus (31)	49758.40 + 8762.13 (47992.16)	50778.00 + 8413.47 (46082.47)	33679.60 + 7209.15 (39486.14)	16681.53 + 6334.71 (34696.63)	4908.93 + 1725.57 (9451.33)	< 0.001**
Left G_temp_sup-G_T_transv (33)	44577.47 + 7192.04 (39392.42)	22894.47 + 2812.57 (15405.09)	18342.73 + 3178.58 (17409.82)	9582.40 + 1155.84 (6330.82)	979.20 + 410.38 (2247.76)	< 0.001**
Left G_temporal_middle (38)	146013.60 + 23001.56 (125984.76)	110814.07 + 18063.21 (98936.26)	58867.87 + 9263.71 (50739.42)	40733.87 + 7749.29 (42444.63)	19833.53 + 4697.76 (25730.68)	< 0.001**
Left Lat_Fis-ant-Vertical (40)	15182.00 + 4762.28 (26084.08)	26746.73 + 5200.90 (28486.48)	17831.87 + 3012.40 (16499.58)	12839.00 + 1971.15 (10796.44)	1397.93 + 621.08 (3401.82)	0.0059**
Left S_cingul-Marginalis (46)	35560.93 + 7747.29 (42433.66)	87018.40 + 16690.23 (91416.14)	62532.53 + 7693.19 (42137.34)	31877.67 + 6749.48 (36968.45)	5717.47 + 1707.92 (9354.67)	< 0.001**
Left S_circular_insula_ant (47)	22985.20 + 4640.74 (25418.36)	35558.53 + 5096.53 (27914.86)	25872.33 + 4116.32 (22546.02)	14273.87 + 3854.08 (21109.66)	3291.47 + 1474.28 (8074.96)	< 0.001**
Left S_circular_insula_sup (49)	45641.20 + 13876.54 (76004.93)	114592.33 + 17161.34 (93996.54)	100037.20 + 15239.67 (83471.11)	39018.87 + 7610.79 (41685.99)	7777.07 + 3736.63 (20466.35)	< 0.001**
Left S_front_inf (52)	46978.00 + 7706.07 (42207.87)	73656.53 + 7682.63 (42079.50)	56670.33 + 7440.35 (40752.50)	36598.60 + 4754.43 (26041.10)	15051.80 + 3372.90 (18474.13)	< 0.001**
Left S_occipital_ant (59)	16028.07 + 4141.87 (22685.95)	28534.40 + 5201.06 (28487.36)	29355.47 + 4815.49 (26375.50)	12761.00 + 1635.56 (8958.32)	6905.60 + 2521.90 (13813.04)	0.0083**
Left S_oc-temp_med_and_Lingual (61)	54830.93 + 11201.79 (61354.72)	93648.20 + 16667.08 (91289.37)	64551.60 + 15222.25 (83375.67)	28342.80 + 6541.65 (35830.07)	11213.13 + 3685.45 (20186.04)	0.0018**
Left S_orbital_med-olfact (63)	672.00 + 443.23 (2427.68)	14931.47 + 2050.08 (11228.73)	19011.33 + 2960.52 (16215.42)	13536.13 + 2785.66 (15257.66)	1973.20 + 1862.66 (10202.20)	< 0.001**
Left S_suborbital (70)	12237.33 + 3363.73 (18423.92)	26963.20 + 4342.88 (23786.93)	34860.93 + 5771.77 (31613.27)	25693.13 + 4452.08 (24385.07)	8704.27 + 2521.95 (13813.26)	0.0060**
Left S_temporal_transverse (74)	11475.07 + 3275.49 (17940.62)	24588.33 + 3801.26 (20820.34)	21991.53 + 3033.11 (16613.03)	12302.00 + 2367.54 (12967.55)	2215.53 + 1078.86 (5909.19)	< 0.001**
Right G_and_S_cingul-Ant (80)	55586.93 + 9315.19 (51021.40)	74826.00 + 9236.73 (50591.64)	47476.27 + 8035.36 (44011.45)	18669.27 + 2788.06 (15270.83)	9350.33 + 2283.61 (12507.86)	< 0.001**
Right G_and_S_cingul-Mid-Ant (81)	65371.73 + 8939.97 (48966.23)	74850.20 + 10919.41 (59808.07)	39805.93 + 6423.38 (35182.32)	25395.40 + 4188.51 (22941.44)	10791.80 + 2974.46 (16291.80)	< 0.001**
Right G_and_S_cingul-Mid-Post (82)	113381.33 + 29726.58 (162819.16)	122612.87 + 20248.73 (110906.88)	74585.27 + 13976.73 (76553.69)	34606.20 + 7473.26 (40932.72)	17887.00 + 4195.16 (22977.81)	0.0041**
Right G_cingul-Post-dorsal (83)	23924.87 + 4250.26 (23279.61)	23338.00 + 4047.51 (22169.10)	16097.13 + 3797.57 (20800.12)	9110.20 + 3240.15 (17747.05)	3210.33 + 2138.55 (11713.33)	0.0092**

Right G_cingul-Post-ventral (84)	28772.07 + 6950.96 (38071.97)	32604.80 + 5563.76 (30473.95)	19917.33 + 2536.21 (13891.39)	13543.93 + 1749.60 (9582.93)	6.13 + 4.67 (25.57)	< 0.001**
Right G_front_inf-Opercular (86)	114503.87 + 14029.92 (76845.05)	50750.13 + 7017.15 (38434.53)	23137.67 + 3752.09 (20551.06)	12887.47 + 3035.17 (16624.30)	2561.27 + 960.13 (5258.86)	< 0.001**
Right G_front_inf-Orbital (87)	32034.60 + 6652.63 (36437.97)	29211.07 + 4774.17 (26149.18)	11475.40 + 1574.31 (8622.87)	8079.73 + 1524.26 (8348.73)	815.07 + 311.56 (1706.51)	< 0.001**
Right G_front_inf-Triangul (88)	40368.67 + 6081.42 (33309.30)	31831.73 + 5742.61 (31453.58)	23557.00 + 4908.63 (26885.65)	14391.67 + 1873.30 (10260.50)	8488.27 + 2697.02 (14772.17)	< 0.001**
Right G_front_middle (89)	148545.87 + 14683.05 (80422.35)	146963.40 + 14169.56 (77609.90)	98000.87 + 11164.55 (61150.78)	61967.33 + 7004.93 (38367.58)	24693.67 + 3439.13 (18836.87)	< 0.001**
Right G_front_sup (90)	247404.40 + 37106.55 (203240.96)	218354.00 + 36669.06 (200844.69)	134093.00 + 17285.60 (94677.11)	89596.73 + 13359.44 (73172.65)	68420.93 + 18488.22 (101264.14)	< 0.001**
Right G_insular_short (92)	34237.87 + 9422.57 (51609.53)	17261.93 + 3217.12 (17620.89)	10250.00 + 1653.84 (9058.43)	6844.20 + 1426.84 (7815.10)	847.47 + 520.46 (2850.69)	< 0.001**
Right G_occipital_middle (93)	124539.47 + 23477.59 (128592.04)	106470.73 + 11727.07 (64231.80)	73541.20 + 10317.94 (56513.66)	45579.60 + 7462.79 (40875.40)	17482.53 + 4823.35 (26418.56)	< 0.001**
Right G_oc-temp_lat-fusifor (95)	75604.20 + 16044.66 (87880.21)	66715.80 + 11888.84 (65117.87)	49830.67 + 13981.05 (76577.37)	15404.20 + 3733.42 (20448.81)	1206.93 + 366.23 (2005.94)	< 0.001**
Right G_pariet_inf-Supramar (100)	79013.33 + 13253.20 (72590.78)	73896.33 + 10674.04 (58464.15)	41266.40 + 6132.62 (33589.76)	28127.73 + 5217.09 (28575.20)	13023.40 + 3732.64 (20444.50)	< 0.001**
Right G_temp_sup-G_T_transv (107)	28688.27 + 4889.35 (26780.07)	20297.13 + 2232.21 (12226.34)	17430.13 + 2489.94 (13637.99)	14177.80 + 2140.06 (11721.61)	504.13 + 352.50 (1930.71)	< 0.001**
Right G_temp_sup-Lateral (108)	95378.27 + 12848.63 (70374.86)	53867.53 + 8279.99 (45351.38)	31076.40 + 7279.92 (39873.75)	12418.07 + 2668.68 (14616.96)	4733.20 + 1718.23 (9411.12)	< 0.001**
Right G_temp_sup-Plan_polar (109)	21923.07 + 6344.25 (34748.89)	45282.73 + 8844.36 (48442.56)	40286.27 + 7278.42 (39865.53)	22238.73 + 4242.92 (23239.45)	1997.93 + 975.54 (5343.23)	0.0011**
Right G_temp_sup-Plan_tempo (110)	33089.20 + 5319.40 (29135.57)	31309.07 + 3734.48 (20454.60)	19680.73 + 2898.01 (15873.04)	10149.47 + 1575.88 (8631.48)	2004.87 + 653.60 (3579.92)	< 0.001**
Right G_temporal_inf (111)	69143.53 + 9399.90 (51485.38)	42634.33 + 6911.29 (37854.70)	24553.87 + 4122.87 (22581.91)	16396.87 + 2903.24 (15901.67)	7131.87 + 2271.50 (12441.52)	< 0.001**
Right G_temporal_middle (112)	131183.80 + 19611.96 (107419.13)	84549.53 + 11877.18 (65053.99)	43504.47 + 8081.22 (44262.65)	30459.13 + 6207.62 (34000.54)	8839.93 + 3362.81 (18418.85)	< 0.001**
Right Lat_Fis-ant-Horizont (113)	17184.80 + 4099.31 (22452.86)	37715.67 + 5027.95 (27539.24)	27527.27 + 3130.95 (17148.95)	16467.27 + 2225.48 (12189.48)	1730.87 + 776.08 (4250.75)	< 0.001**
Right Lat_Fis-ant-Vertical (114)	13647.87 + 3235.10 (17719.40)	28609.60 + 4052.91 (22198.72)	32318.67 + 3634.36 (19906.22)	16874.87 + 2592.08 (14197.38)	720.73 + 400.14 (2191.63)	< 0.001**
Right Lat_Fis-post (115)	83925.53 + 15214.60	168775.27 +	128476.47 +	49067.87 + 16505.06	19625.33 +	< 0.001**

	(83333.80)	27786.47 (152192.79)	24449.97 (133917.97)	(90401.95)	9825.30 (53815.39)	
Right S_cingul-Marginalis (120)	38226.27 + 6712.50 (36765.89)	84931.47 + 11904.38 (65203.00)	70800.67 + 9453.11 (51776.84)	33281.40 + 6280.68 (34400.70)	12601.73 + 4438.10 (24308.46)	< 0.001**
Right S_circular_insula_ant (121)	16236.87 + 3566.65 (19535.36)	32662.53 + 4426.03 (24242.34)	24257.20 + 3856.58 (21123.38)	11537.07 + 1578.84 (8647.64)	2259.47 + 839.51 (4598.21)	< 0.001**
Right S_circular_insula_inf (122)	10513.67 + 3048.31 (16696.26)	31816.20 + 4983.51 (27295.79)	39497.73 + 6151.45 (33692.88)	20505.60 + 3337.07 (18277.89)	8525.53 + 4071.07 (22298.14)	< 0.001**
Right S_circular_insula_sup (123)	42860.60 + 16598.59 (90914.22)	88481.13 + 17289.14 (94696.50)	57945.80 + 6283.53 (34416.30)	20047.27 + 3232.24 (17703.72)	463.67 + 203.18 (1112.89)	< 0.001**
Right S_front_inf (126)	75484.53 + 11028.44 (60405.23)	102940.47 + 13079.21 (71637.78)	58452.93 + 8653.86 (47399.15)	33091.60 + 5159.09 (28257.49)	24088.87 + 5169.06 (28312.09)	< 0.001**
Right S_orbital_lateral (136)	2703.67 + 915.52 (5014.51)	13029.40 + 2699.89 (14787.92)	14988.67 + 2018.57 (11056.15)	13860.60 + 1890.52 (10354.82)	5205.60 + 2013.91 (11030.65)	< 0.001**
Right S_precentral-inf-part (142)	43195.27 + 7178.76 (39319.68)	42715.53 + 6119.43 (33517.52)	21427.00 + 4194.98 (22976.84)	17019.00 + 3368.91 (18452.30)	10655.47 + 2894.40 (15853.27)	< 0.001**
Right S_subparietal (145)	17806.80 + 3214.60 (17607.08)	48180.80 + 6639.12 (36363.95)	60487.53 + 9260.85 (50723.74)	41369.80 + 10265.39 (56225.88)	14559.07 + 4233.04 (23185.34)	< 0.001**
Right S_temporal_transverse (148)	22436.53 + 4766.62 (26107.85)	39922.20 + 7980.10 (43708.80)	28496.27 + 4692.51 (25701.92)	15329.33 + 2070.92 (11342.90)	553.60 + 281.58 (1542.26)	< 0.001**
Clustering coefficient						
Left G_cingul-Post-dorsal (9)	5.11 + 0.21 (1.13)	5.09 + 0.21 (1.14)	5.30 + 0.18 (1.01)	5.61 + 0.20 (1.07)	6.40 + 0.24 (1.30)	0.0052**
Left G_oc-temp_lat-fusifor (21)	5.33 + 0.24 (1.34)	5.47 + 0.23 (1.28)	5.70 + 0.20 (1.09)	6.21 + 0.18 (1.01)	7.17 + 0.26 (1.40)	< 0.001**
Left G_temp_sup-G_T_transv (33)	4.94 + 0.24 (1.32)	5.35 + 0.23 (1.26)	5.61 + 0.26 (1.40)	6.28 + 0.30 (1.65)	7.55 + 0.46 (2.50)	< 0.001**
Left Lat_Fis-post (41)	5.04 + 0.25 (1.37)	4.81 + 0.21 (1.15)	5.09 + 0.23 (1.28)	5.80 + 0.31 (1.70)	6.92 + 0.45 (2.47)	< 0.001**
Left S_circular_insula_sup (49)	5.00 + 0.24 (1.33)	4.52 + 0.20 (1.10)	4.62 + 0.19 (1.02)	5.14 + 0.19 (1.04)	6.11 + 0.24 (1.32)	< 0.001**
Left S_temporal_transverse (74)	5.41 + 0.29 (1.60)	5.14 + 0.25 (1.38)	5.40 + 0.27 (1.48)	6.17 + 0.35 (1.92)	7.36 + 0.47 (2.60)	0.0034**
Right	5.47 + 0.22 (1.22)	5.52 + 0.22 (1.23)	5.88 + 0.28 (1.54)	6.56 + 0.36 (1.98)	7.65 + 0.48 (2.65)	0.0017**

G_and_S_subcentral (78)						
Right G_cingul-Post-dorsal (83)	5.28 + 0.20 (1.08)	5.26 + 0.19 (1.03)	5.38 + 0.17 (0.91)	5.73 + 0.17 (0.93)	6.43 + 0.20 (1.10)	0.0040**
Right G_cingul-Post-ventral (84)	5.26 + 0.20 (1.09)	5.14 + 0.18 (1.01)	5.33 + 0.18 (0.98)	5.62 + 0.27 (1.50)	6.76 + 0.37 (2.05)	0.0058**
Right G_front_inf-Opercular (86)	4.66 + 0.25 (1.40)	5.10 + 0.25 (1.36)	5.69 + 0.28 (1.51)	6.41 + 0.36 (1.95)	7.39 + 0.45 (2.45)	< 0.001**
Right G_front_inf-Orbital (87)	5.23 + 0.22 (1.22)	5.41 + 0.22 (1.21)	6.06 + 0.28 (1.54)	6.59 + 0.37 (2.02)	7.52 + 0.40 (2.17)	< 0.001**
Right G_oc-temp_lat-fusifor (95)	5.11 + 0.21 (1.14)	5.08 + 0.19 (1.02)	5.26 + 0.16 (0.90)	5.81 + 0.15 (0.80)	6.94 + 0.21 (1.16)	< 0.001**
Right G_temp_sup-G_T_transv (107)	4.94 + 0.21 (1.17)	4.98 + 0.23 (1.25)	5.37 + 0.20 (1.12)	5.79 + 0.25 (1.38)	6.96 + 0.39 (2.13)	< 0.001**
Right G_temp_sup-Lateral (108)	4.88 + 0.24 (1.32)	5.11 + 0.24 (1.31)	5.55 + 0.25 (1.39)	6.02 + 0.25 (1.37)	6.77 + 0.29 (1.60)	< 0.001**
Right G_temp_sup-Plan_tempo (110)	5.28 + 0.24 (1.29)	5.18 + 0.22 (1.20)	5.51 + 0.23 (1.25)	6.23 + 0.26 (1.41)	7.39 + 0.36 (1.95)	< 0.001**
Right G_temporal_inf (111)	5.39 + 0.24 (1.31)	5.39 + 0.22 (1.23)	5.67 + 0.21 (1.15)	6.17 + 0.23 (1.25)	6.90 + 0.25 (1.35)	0.0015**
Right Lat_Fis-post (115)	4.83 + 0.23 (1.25)	4.44 + 0.18 (0.96)	4.60 + 0.15 (0.84)	5.25 + 0.20 (1.12)	6.27 + 0.29 (1.61)	< 0.001**
Right S_circular_insula_ant (121)	5.94 + 0.28 (1.55)	5.23 + 0.21 (1.16)	5.28 + 0.20 (1.10)	5.89 + 0.23 (1.24)	6.92 + 0.30 (1.64)	0.0018**
Right S_circular_insula_sup (123)	5.02 + 0.22 (1.23)	4.53 + 0.15 (0.80)	4.75 + 0.13 (0.73)	5.62 + 0.22 (1.21)	6.85 + 0.41 (2.24)	< 0.001**
Right S_collat_transv_post (125)	6.02 + 0.21 (1.13)	6.00 + 0.20 (1.08)	6.26 + 0.17 (0.93)	6.68 + 0.20 (1.07)	7.37 + 0.28 (1.53)	0.0030**
Right S_occipital_ant (133)	5.64 + 0.18 (1.00)	5.48 + 0.15 (0.81)	5.62 + 0.17 (0.95)	6.08 + 0.20 (1.08)	6.94 + 0.26 (1.43)	< 0.001**
Right S_oc-temp_med_and_Lingual (135)	5.28 + 0.18 (0.98)	5.12 + 0.18 (0.97)	5.25 + 0.17 (0.93)	5.64 + 0.19 (1.01)	6.37 + 0.25 (1.38)	0.0068**
Participation coefficient						
Left G_cingul-Post-ventral (10)	15.25 + 1.03 (5.65)	15.03 + 1.01 (5.52)	13.11 + 1.07 (5.84)	10.41 + 1.12 (6.15)	7.15 + 1.11 (6.06)	< 0.001**
Left G_oc-temp_med-	14.70 + 1.23 (6.73)	13.68 + 1.20 (6.57)	11.76 + 1.29 (7.04)	8.90 + 1.20 (6.56)	5.39 + 0.96 (5.27)	< 0.001**

Parahip (23)						
Right G_oc-temp_med-Parahip (97)	11.30 + 1.23 (6.74)	10.19 + 1.23 (6.76)	8.30 + 1.15 (6.31)	6.60 + 1.14 (6.26)	3.82 + 0.95 (5.22)	0.0081**
Right G_temp_sup-G_T_transv (107)	12.65 + 1.16 (6.34)	12.04 + 1.19 (6.53)	10.76 + 1.22 (6.68)	8.90 + 1.15 (6.33)	5.24 + 1.03 (5.62)	0.0079**
Right Lat_Fis-ant-Vertical (114)	9.08 + 1.05 (5.75)	9.67 + 1.08 (5.91)	8.60 + 1.04 (5.68)	5.70 + 0.95 (5.21)	3.36 + 0.87 (4.78)	0.0042**
Right S_circular_insula_sup (123)	15.02 + 0.98 (5.36)	15.67 + 1.00 (5.50)	14.40 + 1.12 (6.14)	10.91 + 1.22 (6.68)	7.66 + 1.16 (6.37)	< 0.001**
Right S_temporal_transverse (148)	12.21 + 1.26 (6.88)	12.20 + 1.25 (6.85)	10.39 + 1.18 (6.47)	7.92 + 1.08 (5.94)	5.09 + 1.02 (5.59)	0.0059**

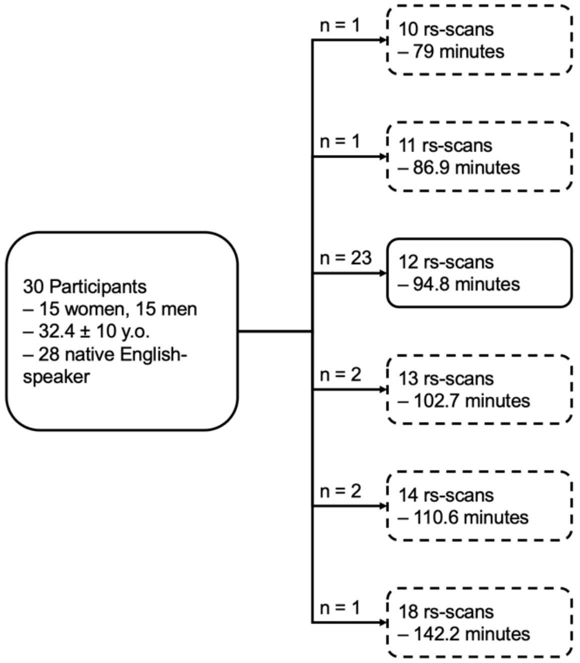

Figure S1. Flowchart of participant scanning organization. The originally designed protocol included 12 resting-state scans (indicated by the solid box).

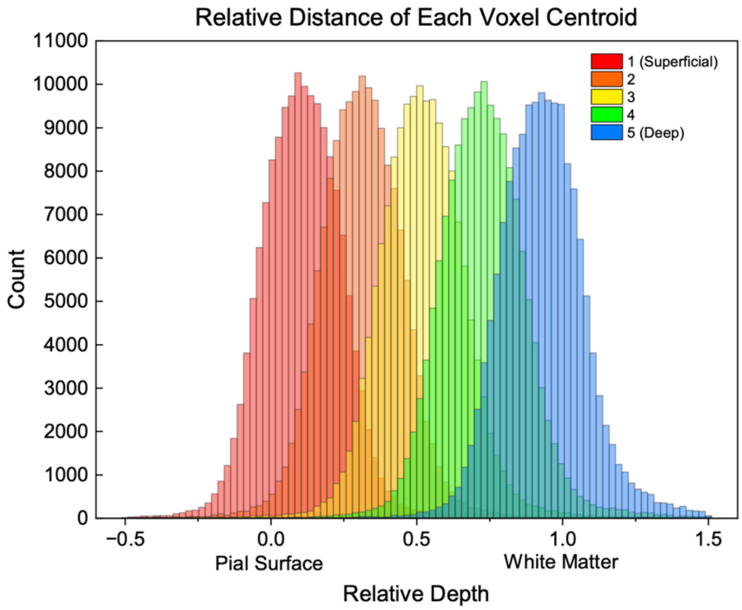

Figure S2. Relative distance of each voxel centroid organized by layer. The distance from each voxel centroid (within cortical volume) to the cortical surface (white matter/pial surface) was calculated. The relative distance was defined so that the depth at pial surface was zero, and one at the white matter border. Next, voxels intersecting each layer were picked and plotted with respect to their relative distances in a histogram.

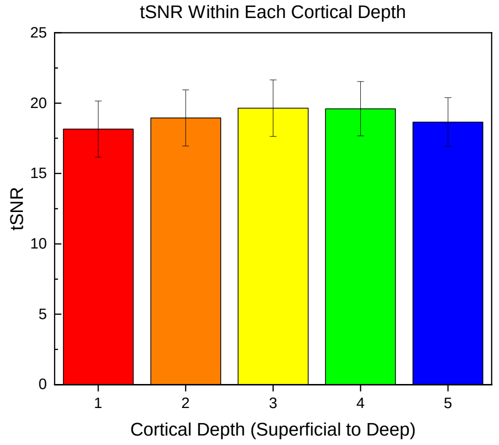

Figure S3. Temporal Signal-to-Noise Ratio (tSNR) across different cortical depths. Error bars indicate standard deviation.

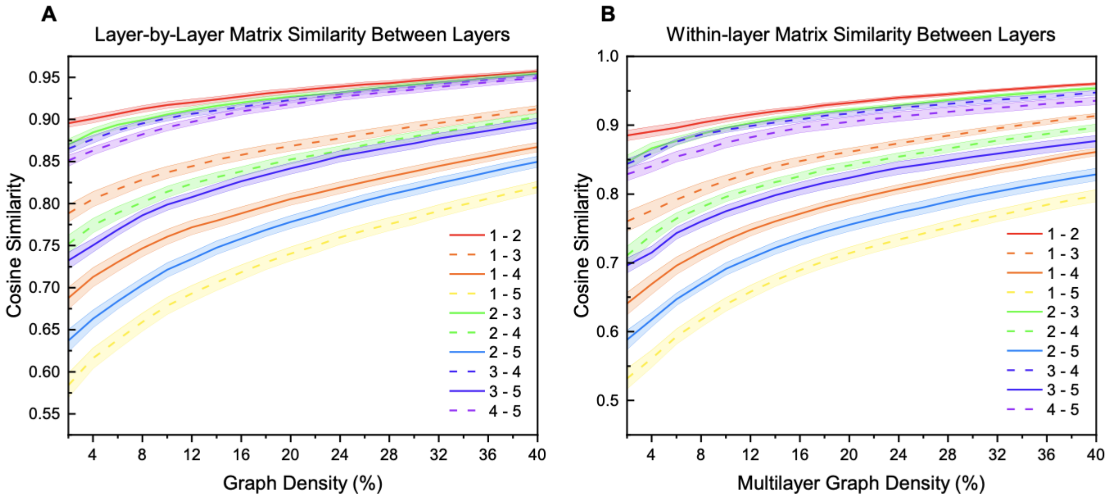

Figure S4. (A) Cosine similarity between each layer using layer-by-layer connectivity matrices. Within participant, each layer's matrix was compared using cosine similarity across a range of thresholds. Cosine similarity values range from -1 (maximal dissimilarity) to +1 (maximal similarity). The mean value at each threshold is plotted while the shaded region indicates the standard error. **(B)** Cosine similarity between each layer using within-layer matrices. With participant, each layer's matrix was compared using cosine similarity across a range of thresholds. The mean value at each threshold is plotted while the shaded region indicates the standard error.

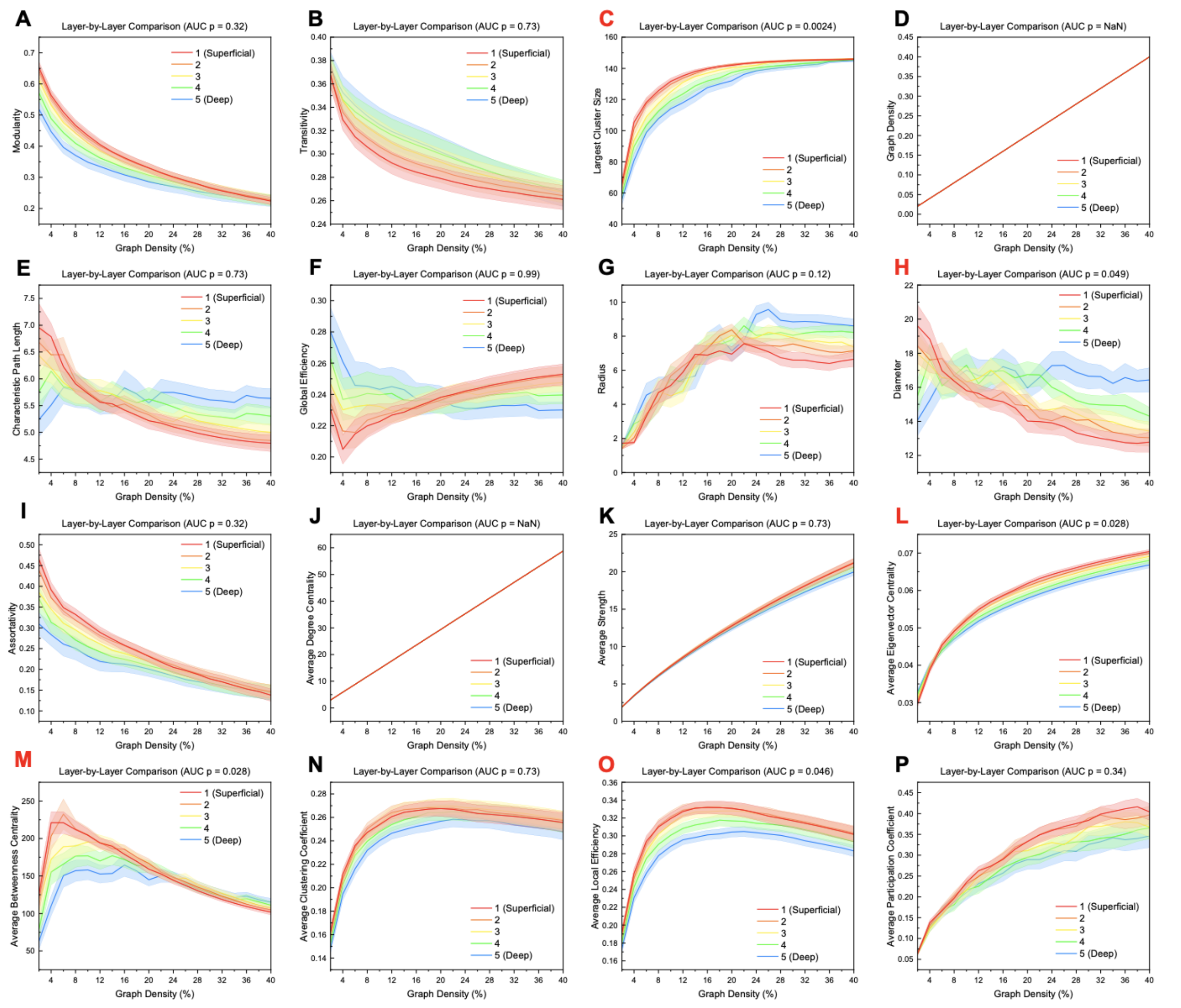

Figure S5. Global measures across different thresholds for layer-by-layer analysis. Significance for area-under-the-curve (AUC) values was calculated using a one-way ANOVA with an FDR correction (alpha = 0.05). Graph density and average degree centrality were constant across layers due to both s being a direct function of thresholding. Red letters indicate a measure that is significantly different between layers.

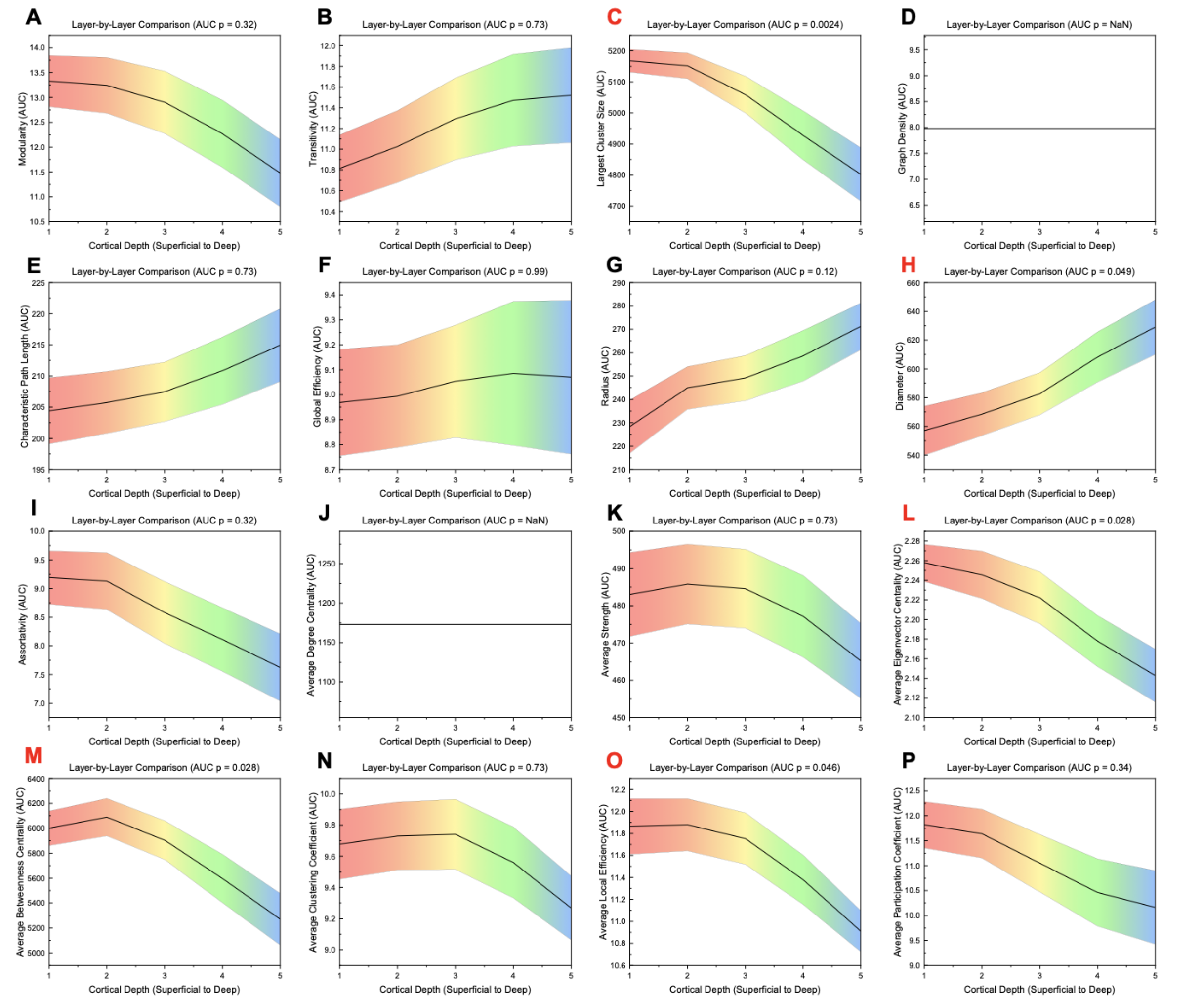

Figure S6. Area-under-the-curve (AUC) values across different layers for global measures for layer-by-layer analysis. Significance was calculated using a one-way ANOVA with an FDR correction (alpha = 0.05). Graph density and average degree centrality were constant across layers due to both s being a direct function of thresholding. Red letters indicate a measure that is significantly different between layers.

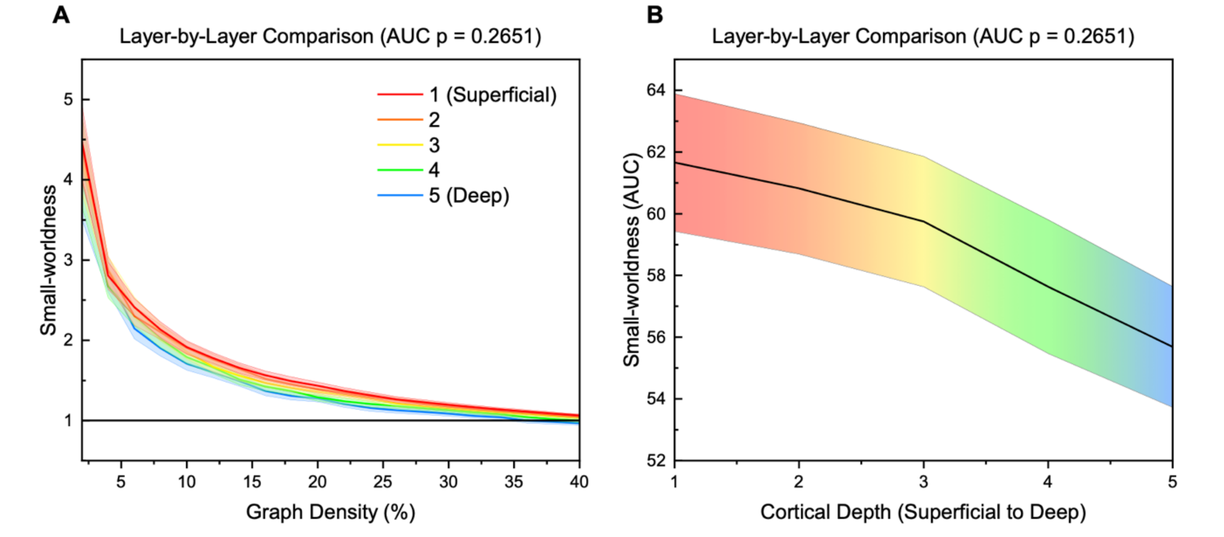

Figure S7. (A) Small-worldness across different thresholds for layer-by-layer analysis. The horizontal black line indicates the small-world threshold of one. **(B)** Area-under-the-curve (AUC) values across different layers for small-worldness for layer-by-layer analysis. Significance for area-under-the-curve (AUC) values was calculated using a one-way ANOVA. The mean value across participants at each layer is plotted while the shaded region indicates the standard error.

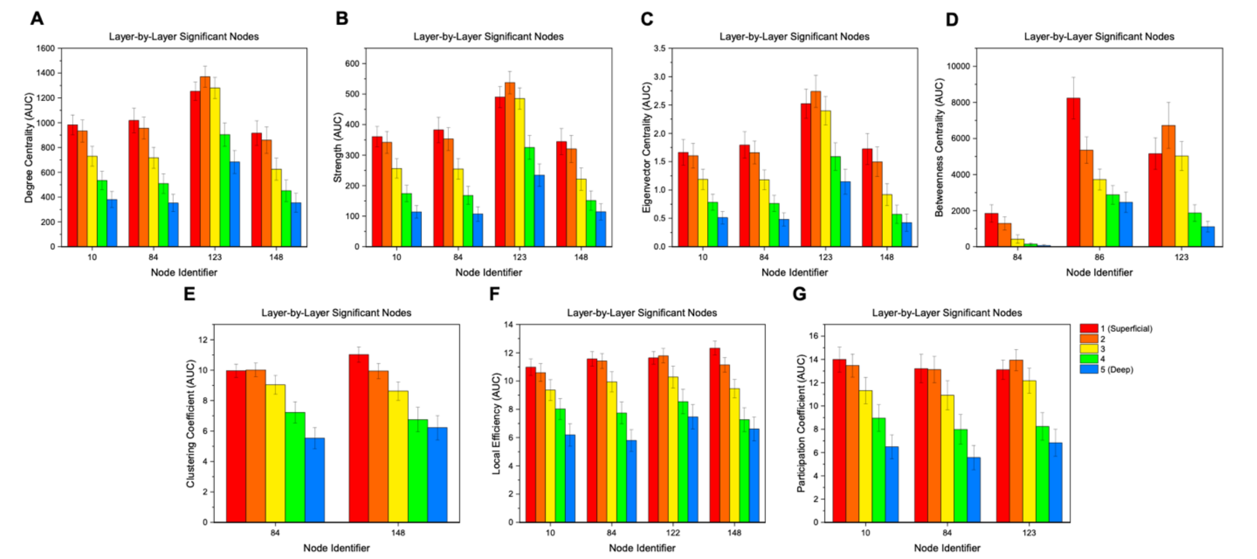

Figure S8. Area-under-the-curve (AUC) values for each significant nodal measure for layer-by-layer analysis: **(A)** degree centrality, **(B)** strength, **(C)** eigenvector centrality, **(D)** betweenness centrality, **(E)** clustering coefficient, **(F)** local efficiency, and **(G)** participation coefficient. Significance was calculated using a one-way ANOVA with an FDR correction (alpha = 0.01) to account for multiple comparisons (Groppe, 2023; Holm, 1979). Error bars display standard error. Node identifier numbers can be found in Table S1.

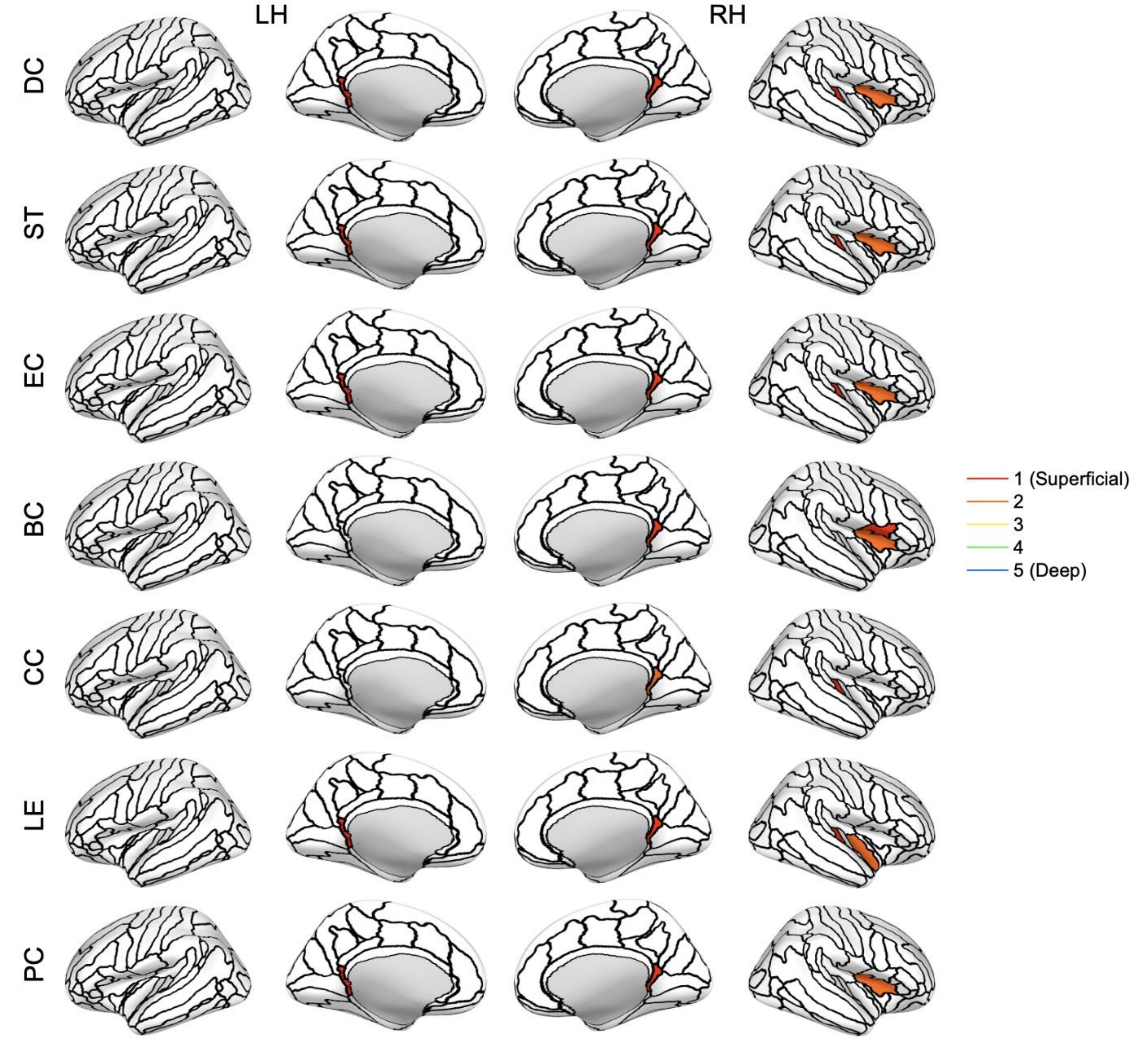

Figure S9. Nodes with significant differences between layers for the layer-by-layer analysis (inflated cortical surface for the corresponding hemisphere). Significance was calculated from the area-under-the curve (AUC) values using a one-way ANOVA with an FDR correction (alpha = 0.01) to account for multiple comparisons (Groppe, 2023; Holm, 1979). The colored section represents the layer with the highest value for the node. The nodes are based on the Destrieux atlas in FreeSurfer (Destrieux et al., 2010; Fischl et al., 2004). LH: left hemisphere; RH: right hemisphere; DC: degree centrality; ST: strength; EC: eigenvector centrality; BC: betweenness centrality; CC: clustering coefficient; LE: local efficiency; PC: participation coefficient.

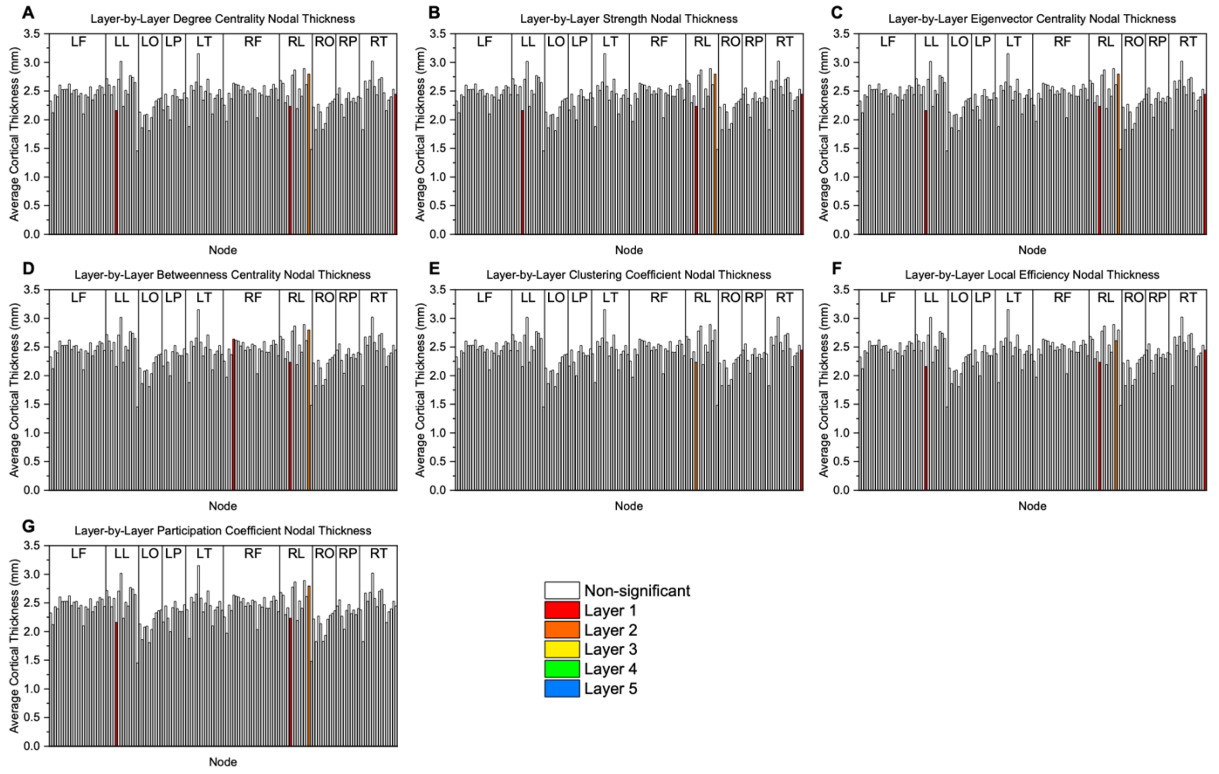

Figure S10. Thickness values grouped by brain region for the layer-by-layer analysis: **(A)** degree centrality, **(B)** strength, **(C)** eigenvector centrality, **(D)** betweenness centrality, **(E)** clustering coefficient, **(F)** local efficiency, and **(G)** participation coefficient. The colored bar represents a significant node and the layer with the highest value for the node. Significance was calculated using a one-way ANOVA with an FDR correction (alpha = 0.01) to account for multiple comparisons (Groppe, 2023; Holm, 1979). LF: left frontal; LL: left limbic; LO: left occipital; LP: left parietal; LT: left temporal; RF: right frontal; RL: right limbic; RO: right occipital; RP: right parietal; RT: right temporal.

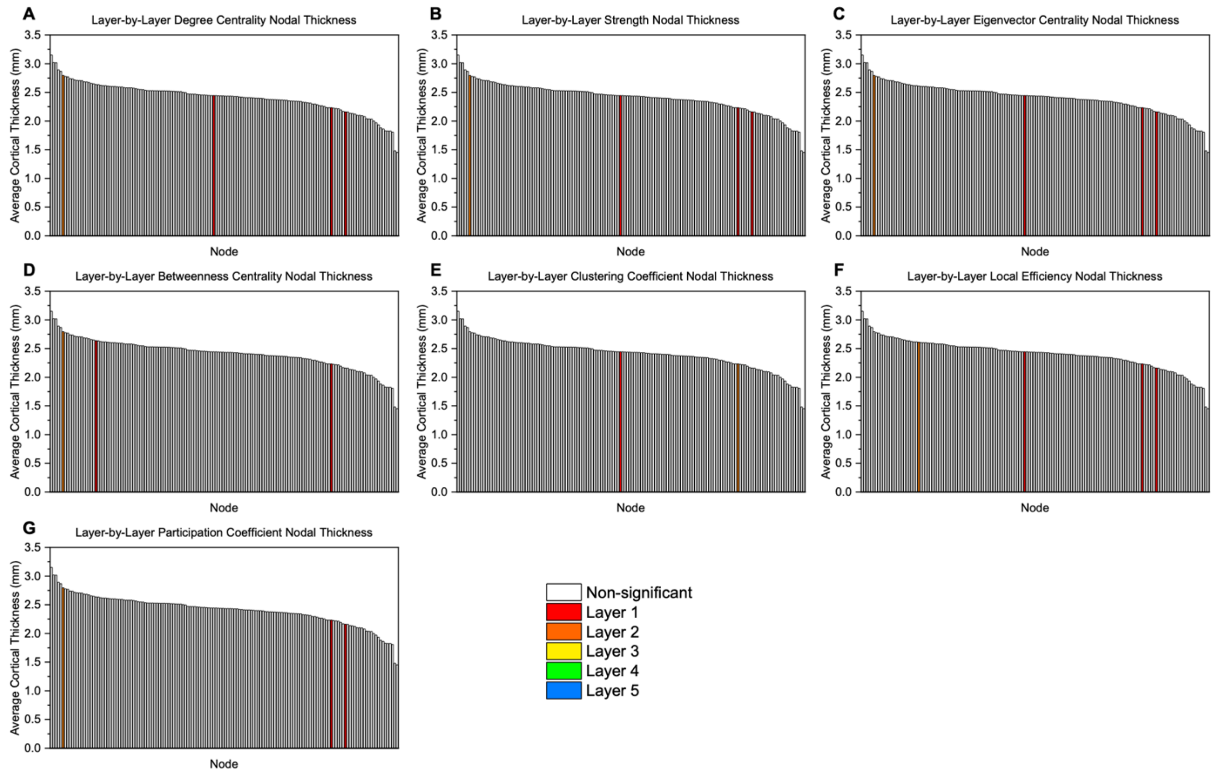

Figure S11. Thickness values sorted from largest to smallest value for the layer-by-layer analysis: **(A)** degree centrality, **(B)** strength, **(C)** eigenvector centrality, **(D)** betweenness centrality, **(E)** clustering coefficient, **(F)** local efficiency, and **(G)** participation coefficient. The colored bar represents a significant node and the layer with the highest value for the node. Significance was calculated using a one-way ANOVA with an FDR correction (alpha = 0.01) to account for multiple comparisons (Groppe, 2023; Holm, 1979). LF: left frontal; LL: left limbic; LO: left occipital; LP: left parietal; LT: left temporal; RF: right frontal; RL: right limbic; RO: right occipital; RP: right parietal; RT: right temporal.

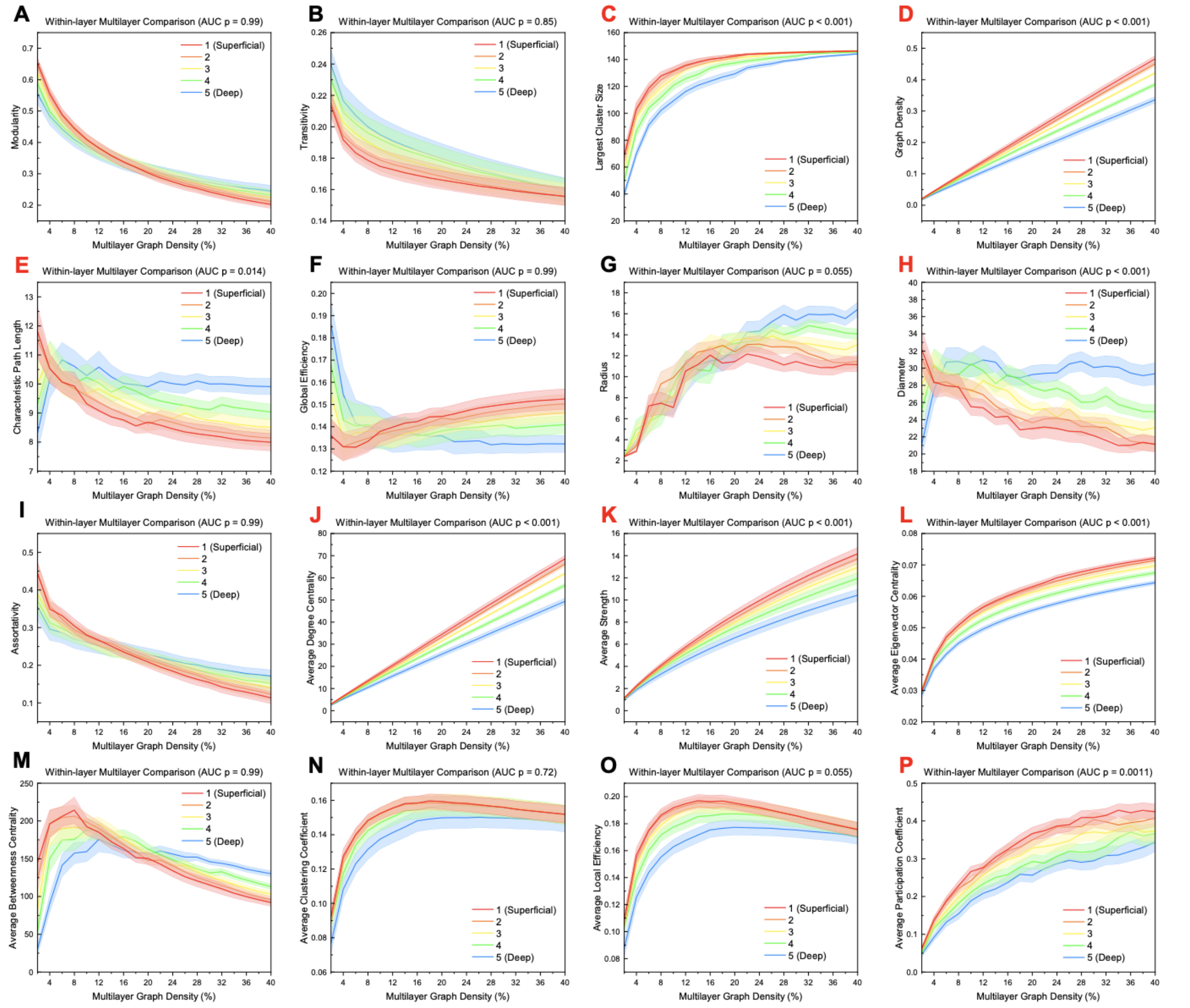

Figure S12. Global measures across different thresholds for within-layer analysis. Significance for area-under-the-curve (AUC) values was calculated using a one-way ANOVA with an FDR correction (alpha = 0.05). Red letters indicate a measure that is significantly different between layers.

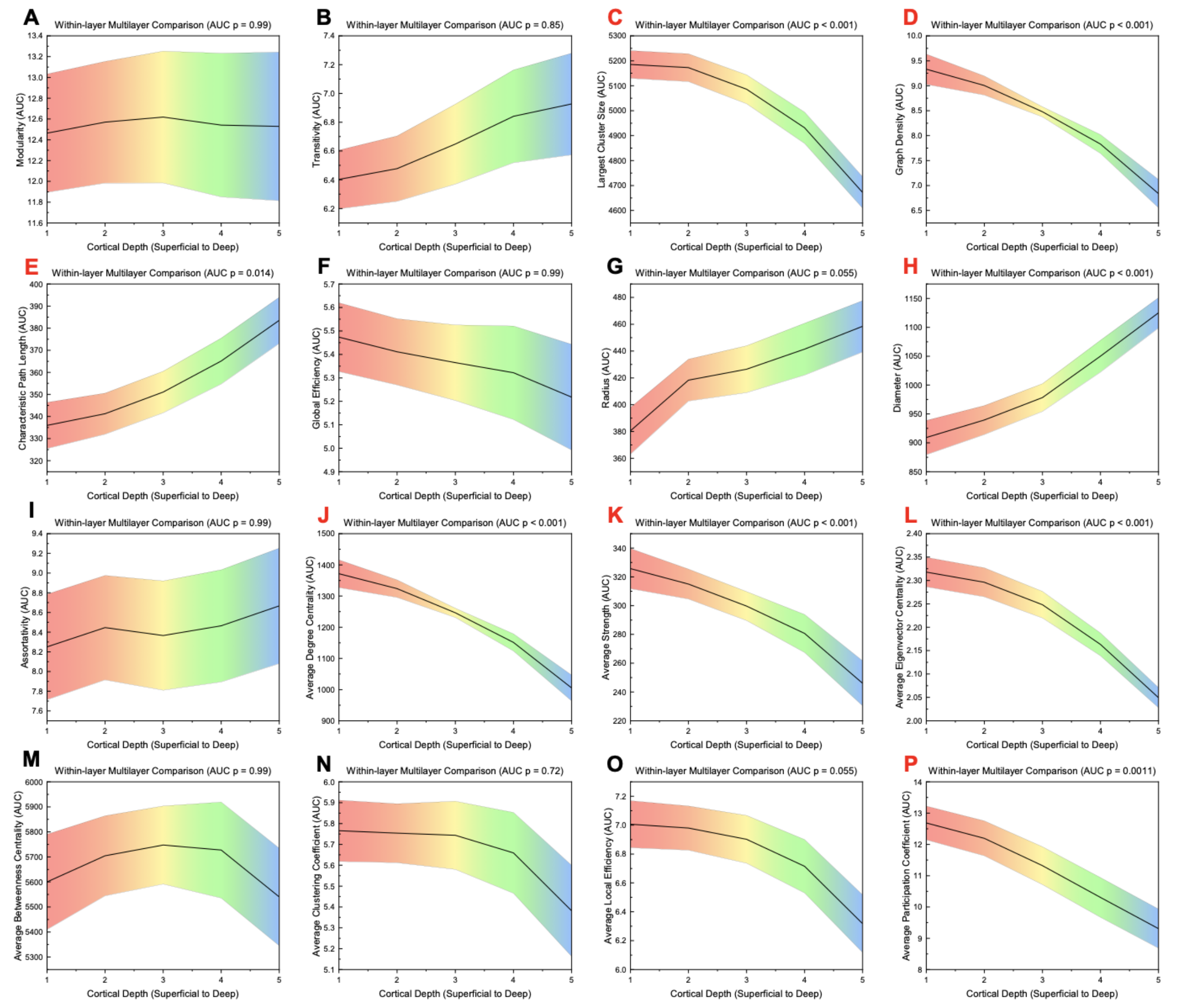

Figure S13. Area-under-the-curve (AUC) values across different layers for global measures for within-layer analysis. Significance was calculated using a one-way ANOVA with an FDR correction (alpha = 0.05). Red letters indicate a measure that is significantly different between layers.

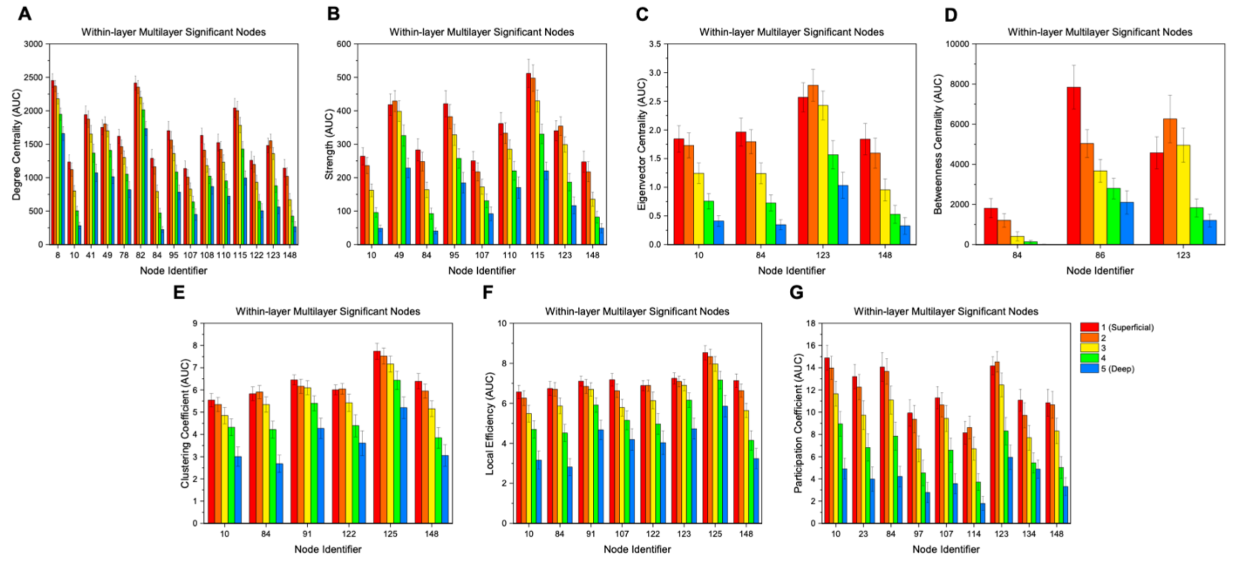

Figure S14. Area-under-the-curve (AUC) values for each significant nodal measure for within-layer analysis: **(A)** degree centrality, **(B)** strength, **(C)** eigenvector centrality, **(D)** betweenness centrality, **(E)** clustering coefficient, **(F)** local efficiency, and **(G)** participation coefficient. Significance was calculated using a one-way ANOVA with an FDR correction (alpha = 0.01) to account for multiple comparisons (Groppe, 2023; Holm, 1979). Error bars display standard error. Node identifier numbers can be found in Table S1.

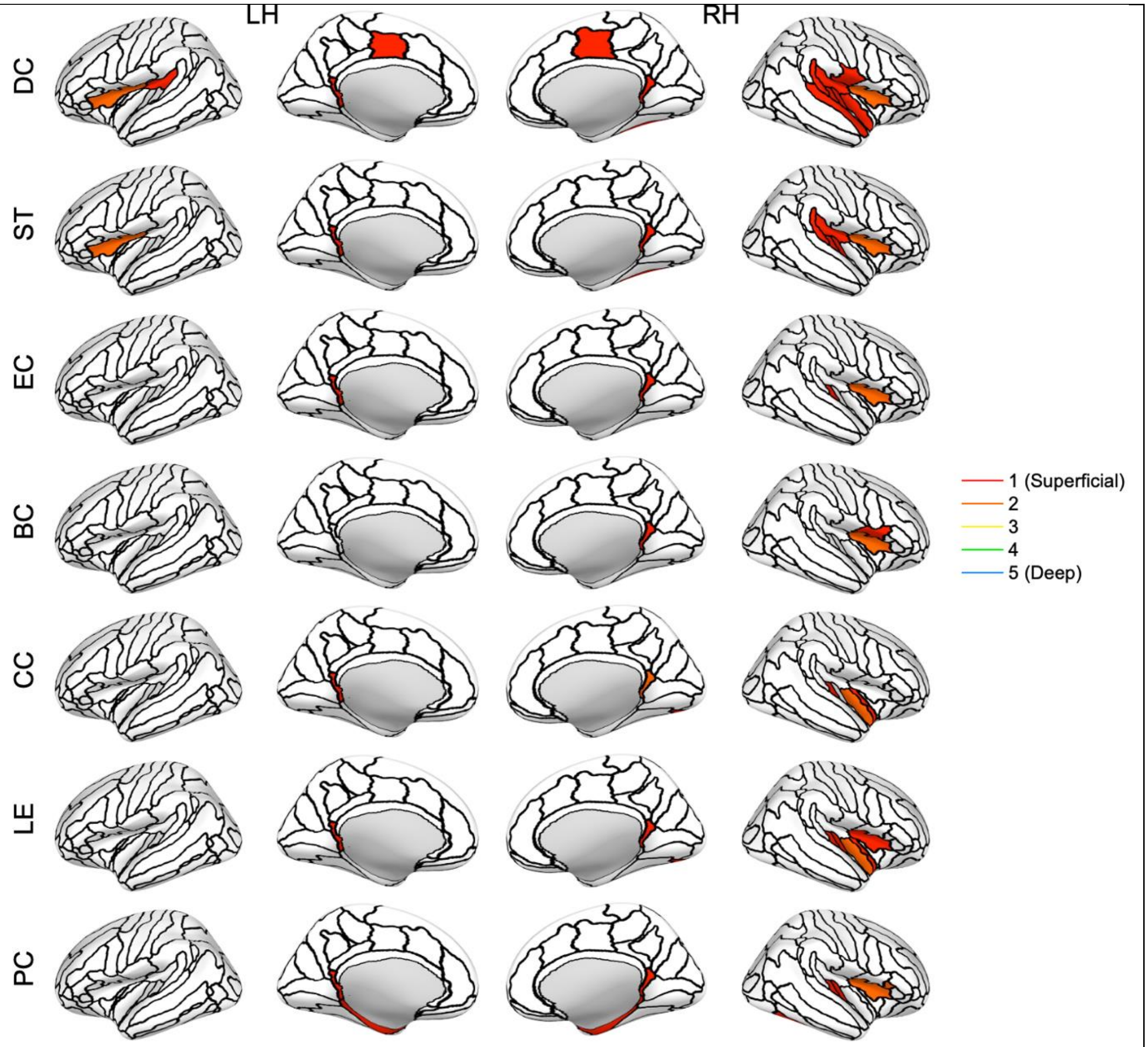

Figure S15. Nodes with significant differences between layers for the within-layer analysis (inflated cortical surface for the corresponding hemisphere). Significance was calculated from the area-under-the curve (AUC) values using a one-way ANOVA with an FDR correction (alpha = 0.01) to account for multiple comparisons (Groppe, 2023; Holm, 1979). The colored section represents the layer with the highest value for the node. The nodes are based on the Destrieux atlas in FreeSurfer (Destrieux et al., 2010; Fischl et al., 2004). LH: left hemisphere; RH: right hemisphere; DC: degree centrality; ST: strength; EC: eigenvector centrality; BC: betweenness centrality; CC: clustering coefficient; LE: local efficiency; PC: participation coefficient.

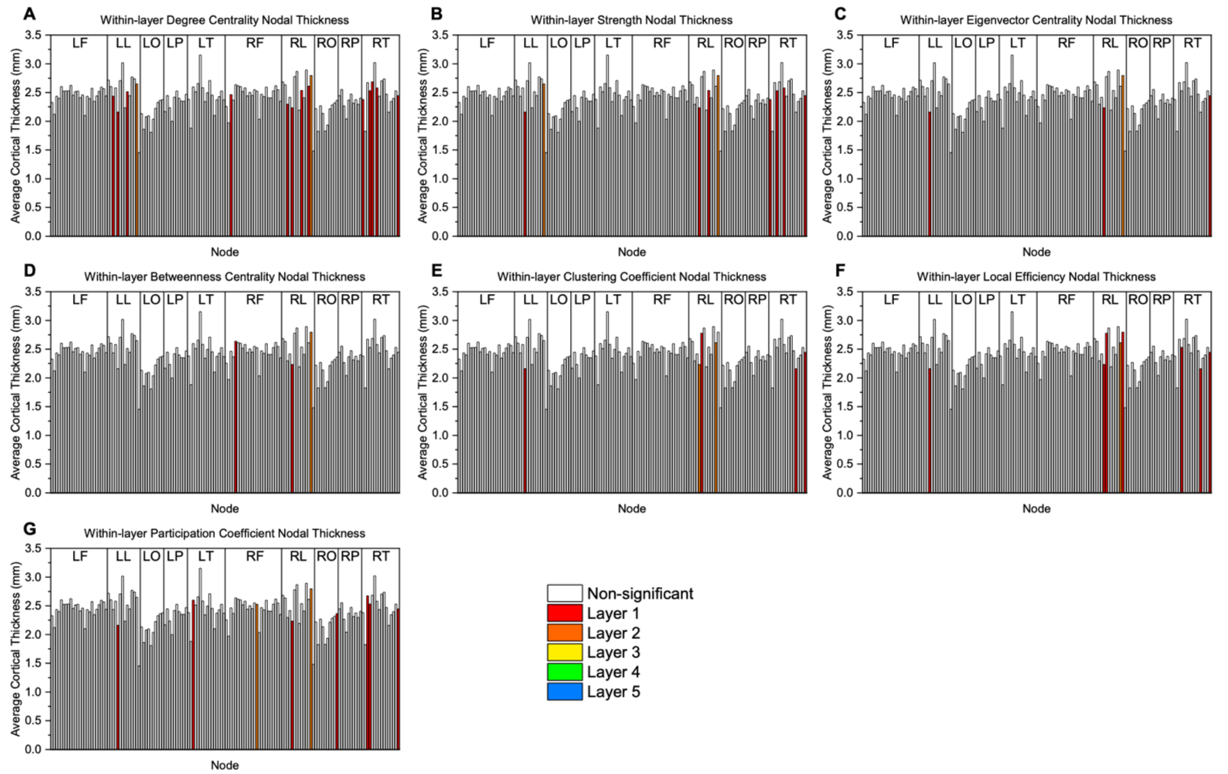

Figure S16. Thickness values grouped by brain region for the within-layer analysis: **(A)** degree centrality, **(B)** strength, **(C)** eigenvector centrality, **(D)** betweenness centrality, **(E)** clustering coefficient, **(F)** local efficiency, and **(G)** participation coefficient. The colored bar represents a significant node and the layer with the highest value for the node. Significance was calculated using a one-way ANOVA with an FDR correction (alpha = 0.01) to account for multiple comparisons (Groppe, 2023; Holm, 1979). LF: left frontal; LL: left limbic; LO: left occipital; LP: left parietal; LT: left temporal; RF: right frontal; RL: right limbic; RO: right occipital; RP: right parietal; RT: right temporal.

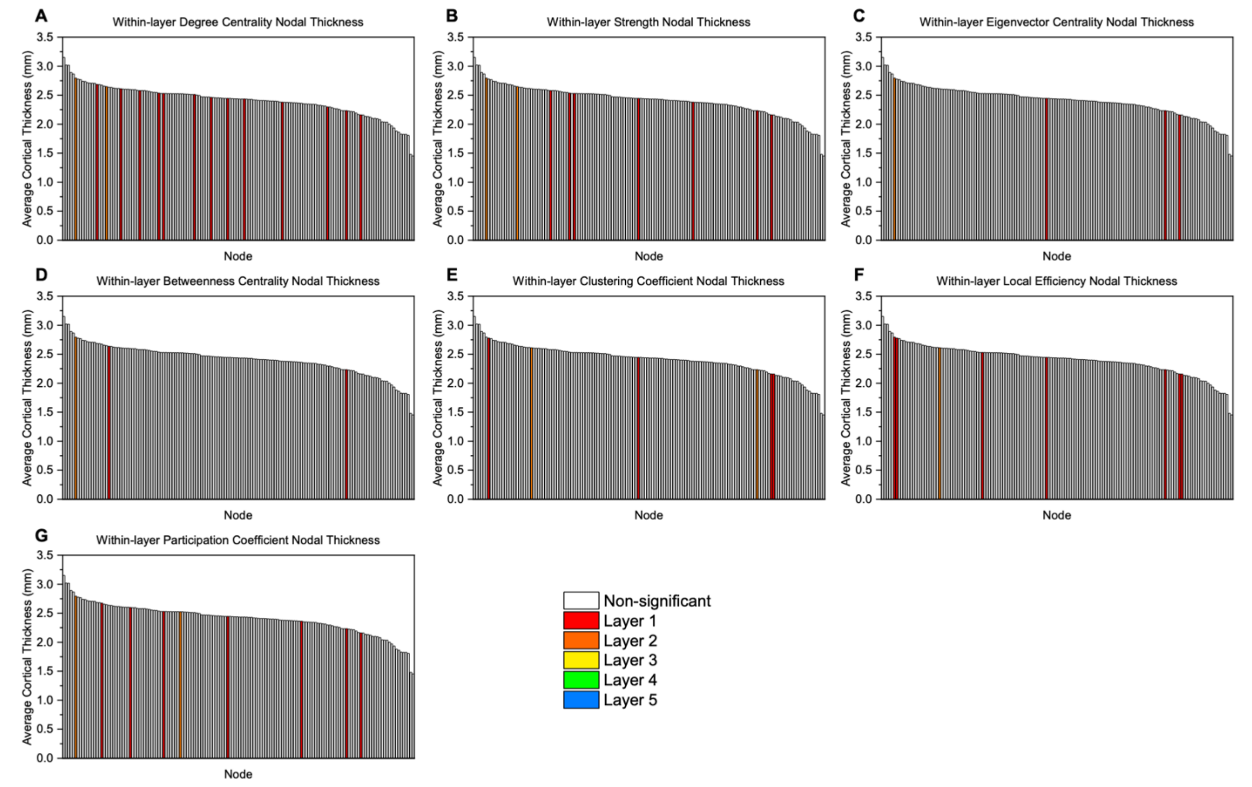

Figure S17. Thickness values sorted from largest to smallest value for the within-layer analysis: **(A)** degree centrality, **(B)** strength, **(C)** eigenvector centrality, **(D)** betweenness centrality, **(E)** clustering coefficient, **(F)** local efficiency, and **(G)** participation coefficient. The colored bar represents a significant node and the layer with the highest value for the node. Significance was calculated using a one-way ANOVA with an FDR correction (alpha = 0.01) to account for multiple comparisons (Groppe, 2023; Holm, 1979). LF: left frontal; LL: left limbic; LO: left occipital; LP: left parietal; LT: left temporal; RF: right frontal; RL: right limbic; RO: right occipital; RP: right parietal; RT: right temporal.

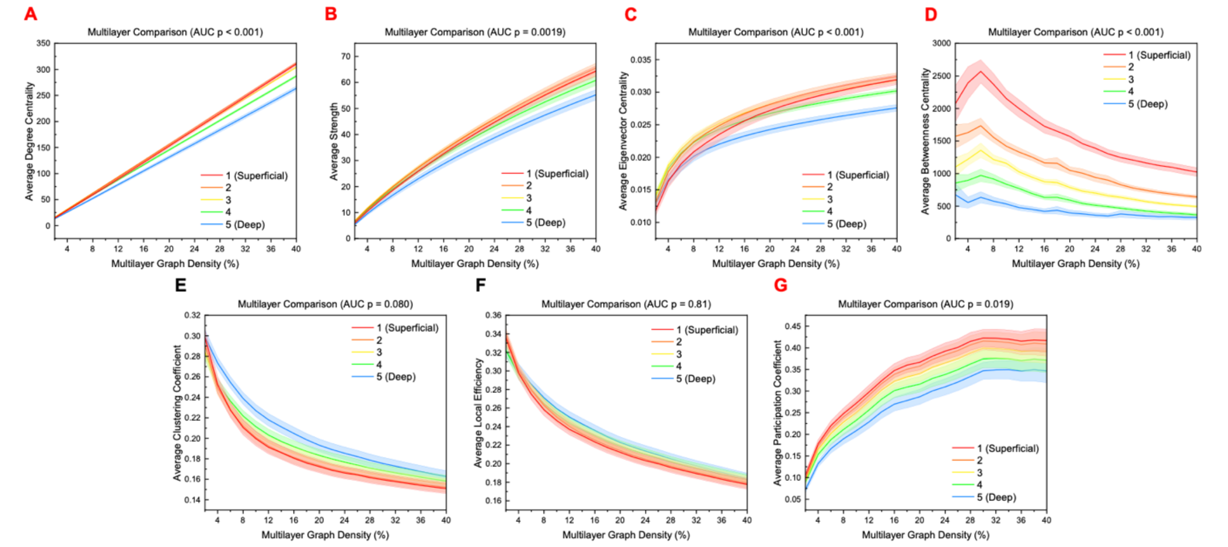

Figure S18. Global measures across different thresholds for multilayer analysis. Significance for area-under-the-curve (AUC) values was calculated using a one-way ANOVA with an FDR correction (alpha = 0.05). Only node-averaged global measures are shown since layer-specific global values cannot be extracted from supra-adjacency matrices. Red letters indicate a measure that is significantly different between layers.

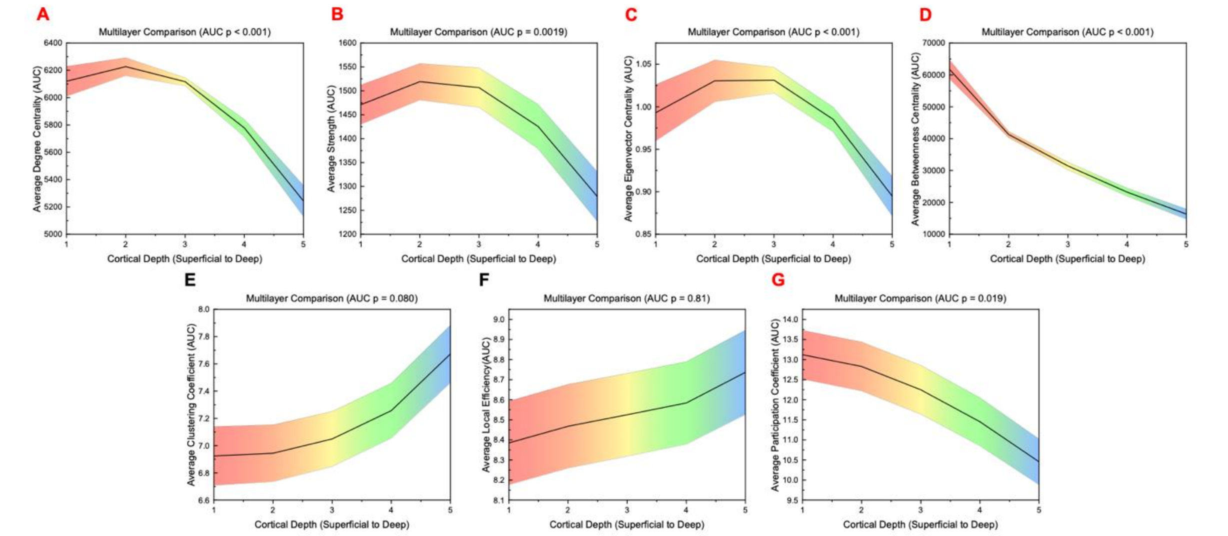

Figure S19. Area-under-the-curve (AUC) values across different layers for global measures for multilayer analysis. Significance was calculated using a one-way ANOVA with an FDR correction (alpha = 0.05). Only node-averaged global measures are shown since layer-specific global values cannot be extracted from supra-adjacency matrices. Red letters indicate a measure that is significantly different between layers.

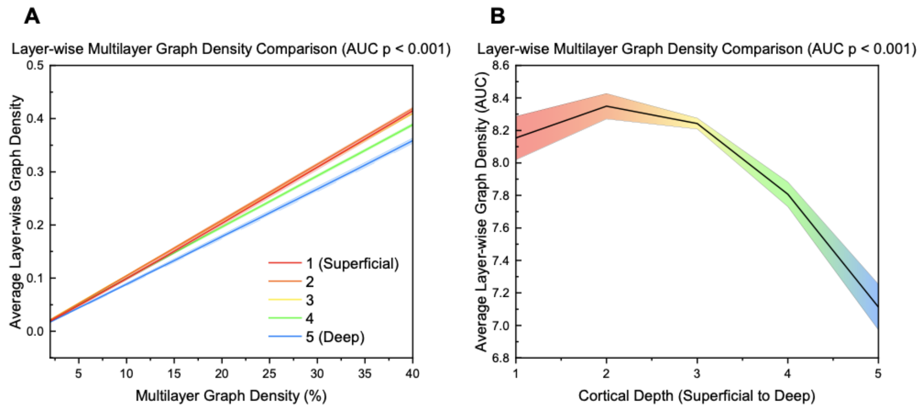

Figure S20. (A) Layer-wise graph density across different thresholds for multilayer analysis. **B)** Area-under-the-curve (AUC) values across different layers for layer-wise graph density for multilayer analysis. Significance for area-under-the-curve (AUC) values was calculated using a one-way ANOVA. The mean value across participants at each layer is plotted while the shaded region indicates the standard error.

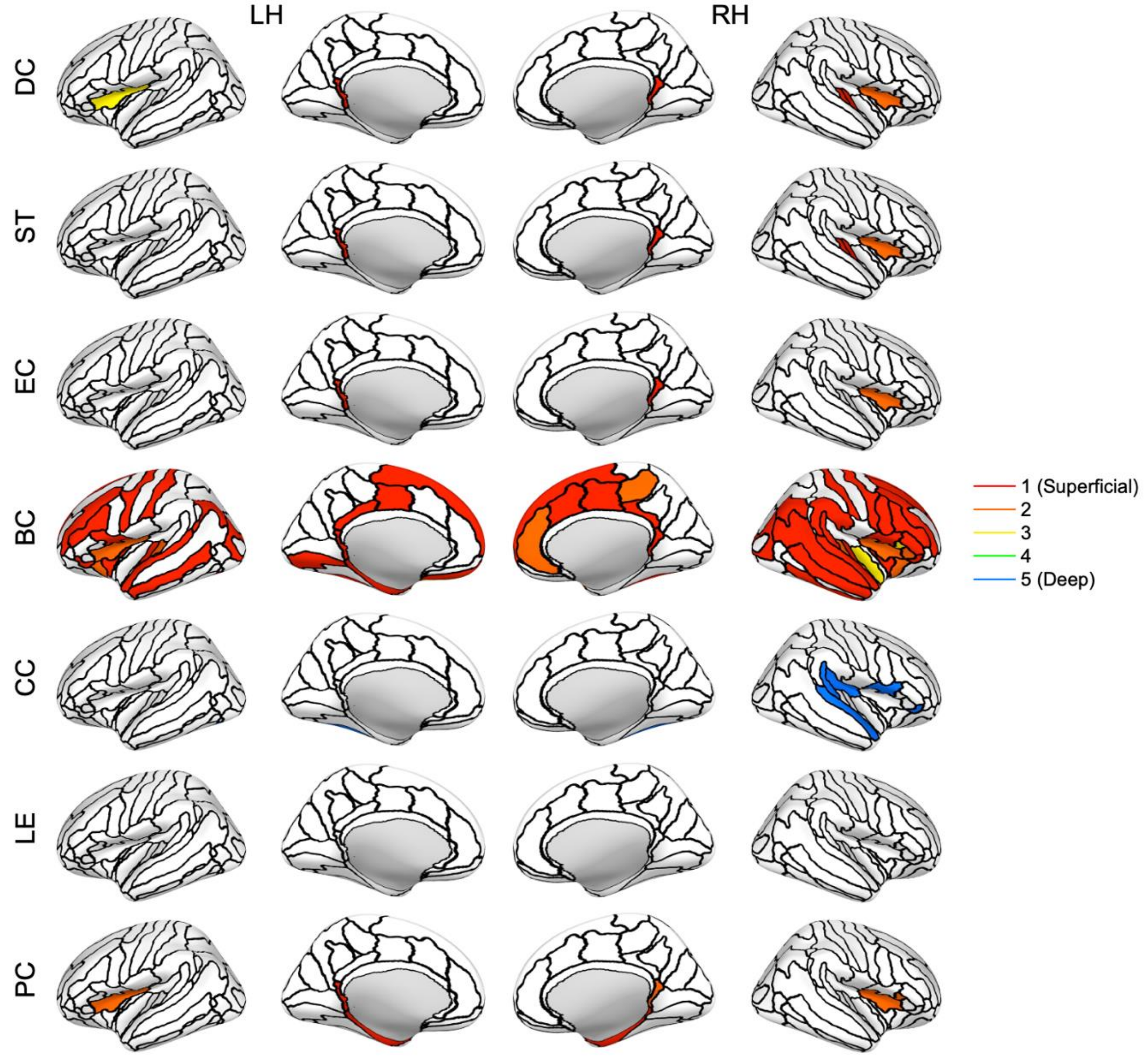

Figure S21. Nodes with significant differences between layers for the multilayer analysis (inflated cortical surface for the corresponding hemisphere). Significance was calculated from the area-under-the curve (AUC) values using a one-way ANOVA with an FDR correction (alpha = 0.01) to account for multiple comparisons (Groppe, 2023; Holm, 1979). The colored section represents the layer with the highest value for the node. The nodes are based on the Destrieux atlas in FreeSurfer (Destrieux et al., 2010; Fischl et al., 2004). LH: left hemisphere; RH: right hemisphere; DC: degree centrality; ST: strength; EC: eigenvector centrality; BC: betweenness centrality; CC: clustering coefficient; LE: local efficiency; PC: participation coefficient.

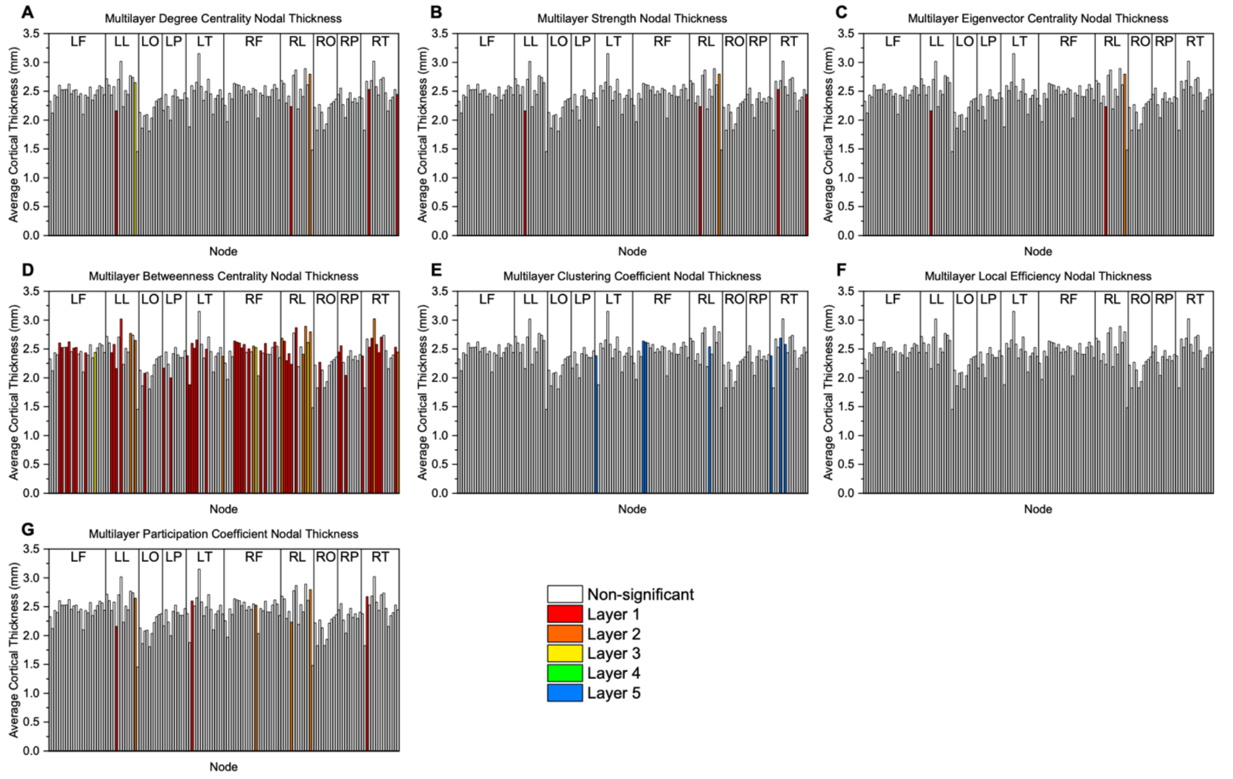

Figure S22. Thickness values grouped by brain region for the multilayer analysis: **(A)** degree centrality, **(B)** strength, **(C)** eigenvector centrality, **(D)** betweenness centrality, **(E)** clustering coefficient, **(F)** local efficiency, and **(G)** participation coefficient. The colored bar represents a significant node and the layer with the highest value for the node. Significance was calculated using a one-way ANOVA with an FDR correction (alpha = 0.01) to account for multiple comparisons (Groppe, 2023; Holm, 1979). LF: left frontal; LL: left limbic; LO: left occipital; LP: left parietal; LT: left temporal; RF: right frontal; RL: right limbic; RO: right occipital; RP: right parietal; RT: right temporal.

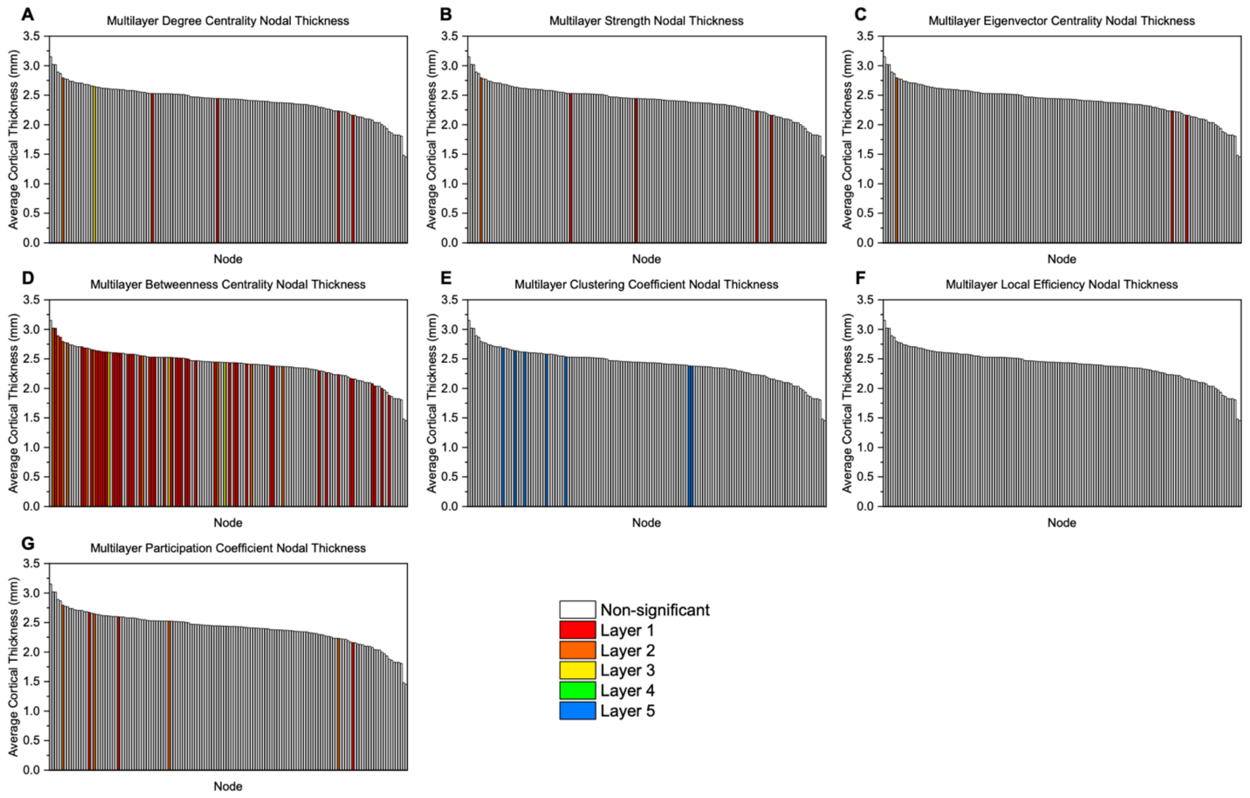

Figure S23. Thickness values sorted from largest to smallest value for the multilayer analysis: **(A)** degree centrality, **(B)** strength, **(C)** eigenvector centrality, **(D)** betweenness centrality, **(E)** clustering coefficient, **(F)** local efficiency, and **(G)** participation coefficient. The colored bar represents a significant node and the layer with the highest value for the node. Significance was calculated using a one-way ANOVA with an FDR correction (alpha = 0.01) to account for multiple comparisons (Groppe, 2023; Holm, 1979). LF: left frontal; LL: left limbic; LO: left occipital; LP: left parietal; LT: left temporal; RF: right frontal; RL: right limbic; RO: right occipital; RP: right parietal; RT: right temporal.

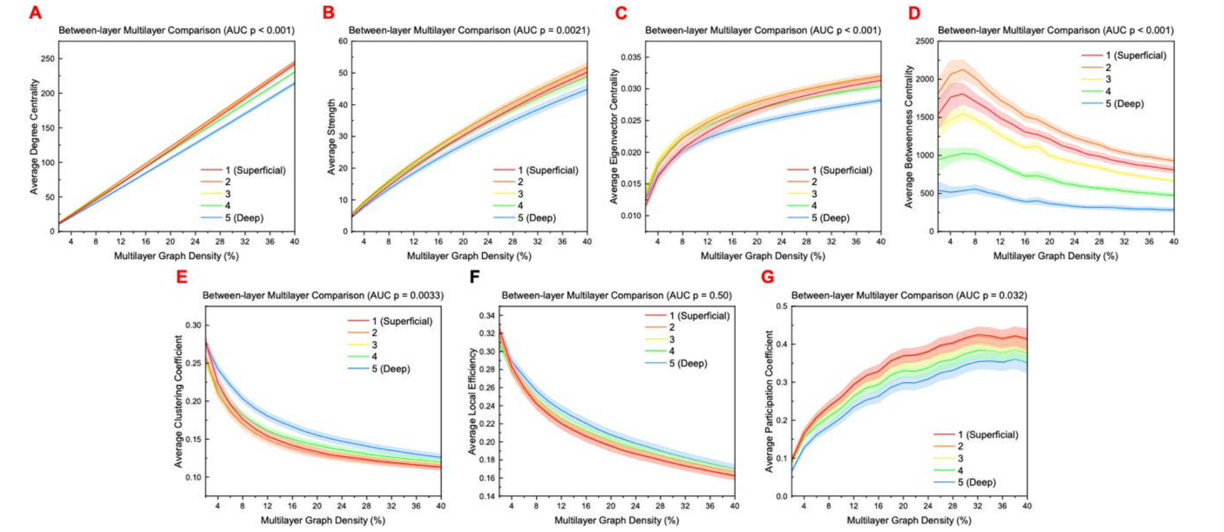

Figure S24. Global measures across different thresholds for between-layer analysis. Significance for area-under-the-curve (AUC) values was calculated using a one-way ANOVA with an FDR correction (alpha = 0.05). Only node-averaged global measures are shown since layer-specific global values cannot be extracted from supra-adjacency matrices. Red letters indicate a measure that is significantly different between layers.

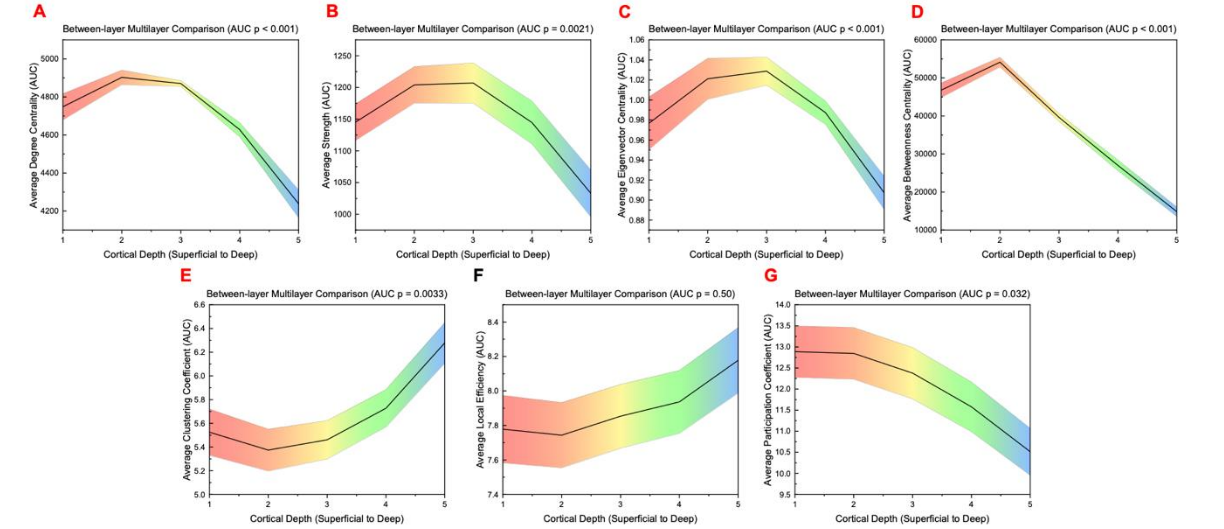

Figure S25. Area-under-the-curve (AUC) values across different layers for global measures for between-layer analysis. Significance was calculated using a one-way ANOVA with an FDR correction (alpha = 0.05). Only node-averaged global measures are shown since layer-specific global values cannot be extracted from supra-adjacency matrices. Red letters indicate a measure that is significantly different between layers.

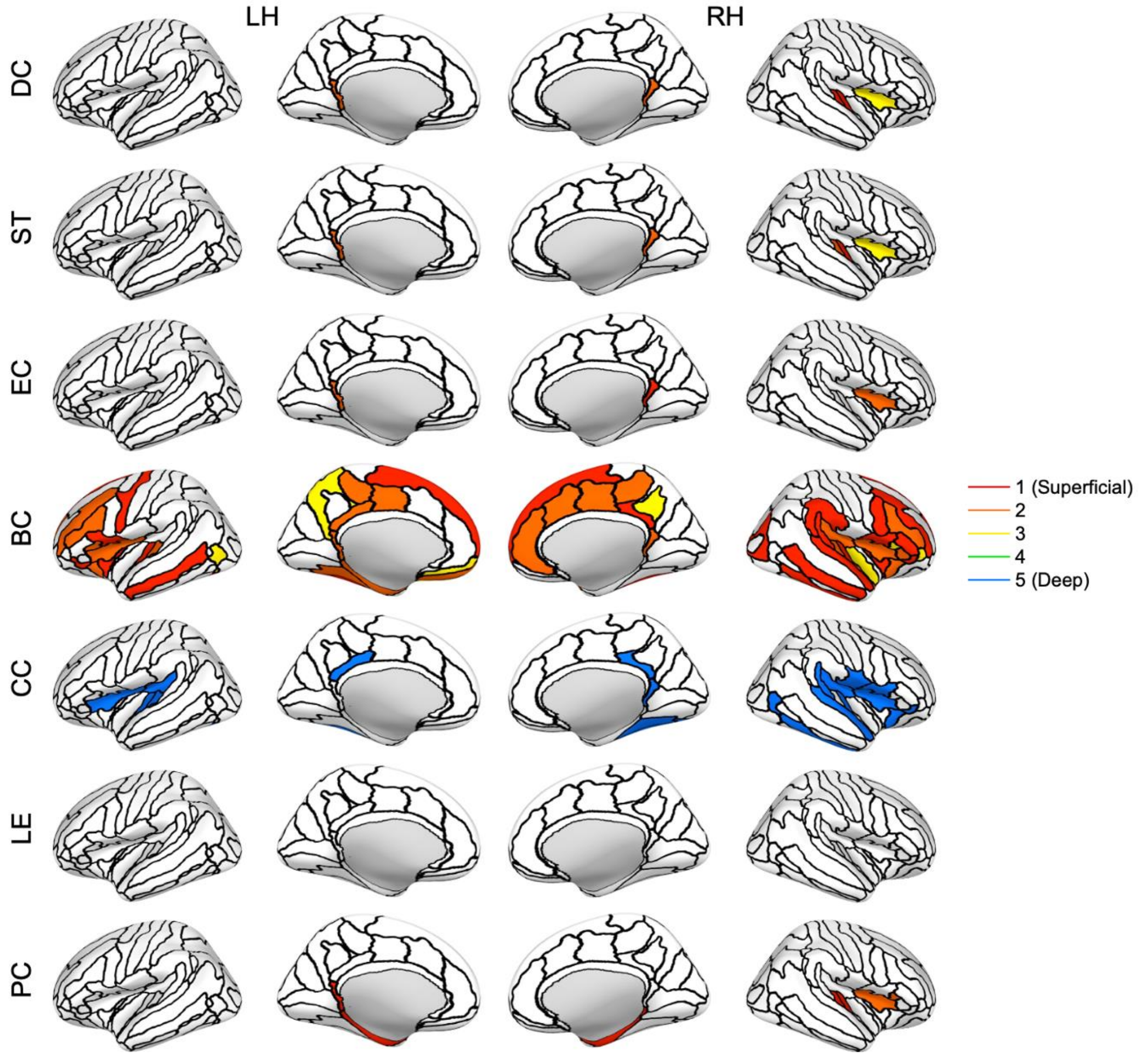

Figure S26. Nodes with significant differences between layers for the between-layer analysis (inflated cortical surface for the corresponding hemisphere). Significance was calculated from the area-under-the curve (AUC) values using a one-way ANOVA with an FDR correction (alpha = 0.01) to account for multiple comparisons (Groppe, 2023; Holm, 1979). The colored section represents the layer with the highest value for the node. The nodes are based on the Destrieux atlas in FreeSurfer (Destrieux et al., 2010; Fischl et al., 2004). LH: left hemisphere; RH: right hemisphere; DC: degree centrality; ST: strength; EC: eigenvector centrality; BC: betweenness centrality; CC: clustering coefficient; LE: local efficiency; PC: participation coefficient.

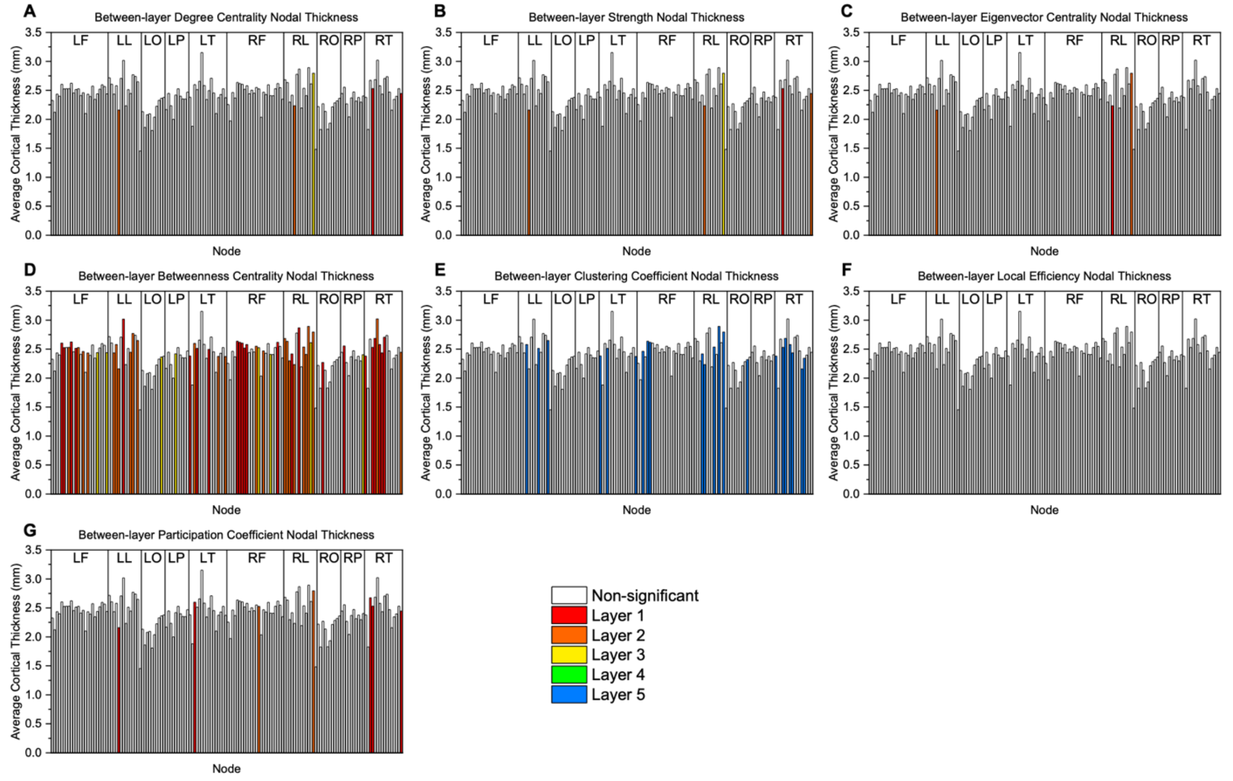

Figure S27. Thickness values grouped by brain region for the between-layer analysis: **(A)** degree centrality, **(B)** strength, **(C)** eigenvector centrality, **(D)** betweenness centrality, **(E)** clustering coefficient, **(F)** local efficiency, and **(G)** participation coefficient. The colored bar represents a significant node and the layer with the highest value for the node. Significance was calculated using a one-way ANOVA with an FDR correction (alpha = 0.01) to account for multiple comparisons (Groppe, 2023; Holm, 1979). LF: left frontal; LL: left limbic; LO: left occipital; LP: left parietal; LT: left temporal; RF: right frontal; RL: right limbic; RO: right occipital; RP: right parietal; RT: right temporal.

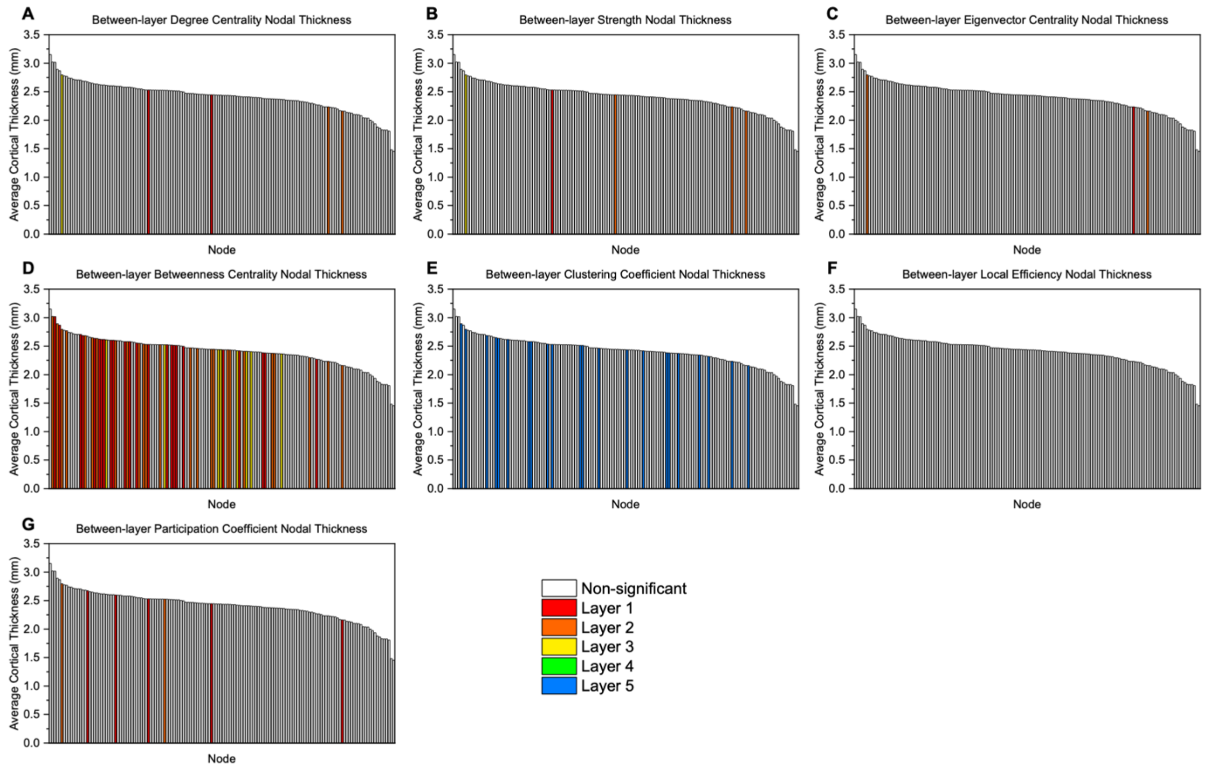

Figure S28. Thickness values sorted from largest to smallest value for the between-layer analysis: **(A)** degree centrality, **(B)** strength, **(C)** eigenvector centrality, **(D)** betweenness centrality, **(E)** clustering coefficient, **(F)** local efficiency, and **(G)** participation coefficient. The colored bar represents a significant node and the layer with the highest value for the node. Significance was calculated using a one-way ANOVA with an FDR correction (alpha = 0.01) to account for multiple comparisons (Groppe, 2023; Holm, 1979). LF: left frontal; LL: left limbic; LO: left occipital; LP: left parietal; LT: left temporal; RF: right frontal; RL: right limbic; RO: right occipital; RP: right parietal; RT: right temporal.

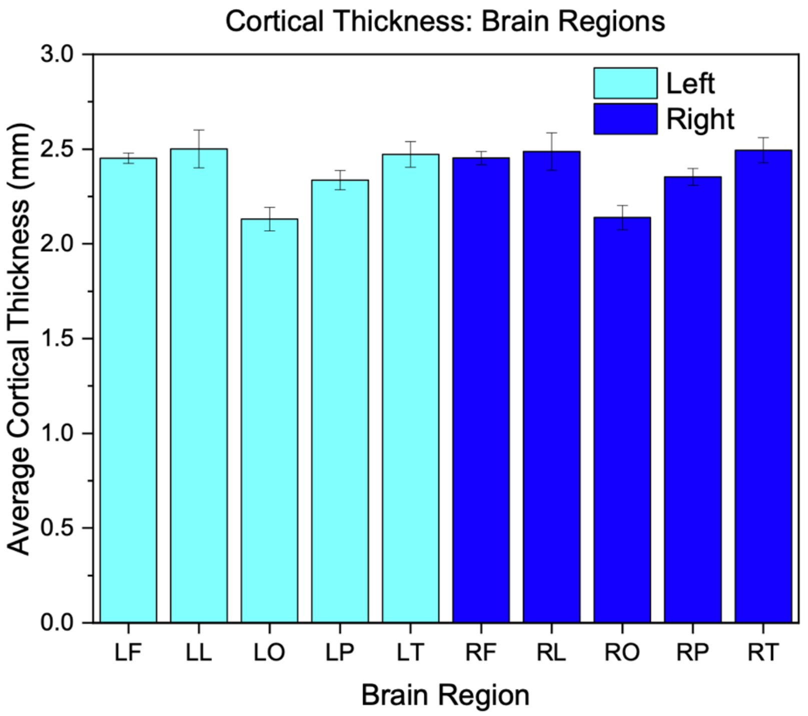

Figure S29. Average cortical thickness for each brain region. Error bars denote standard error. Thickness values are an average across all subjects and all nodes within each region (Table 1). LF: left frontal; LL: left limbic; LO: left occipital; LP: left parietal; LT: left temporal; RF: right frontal; RL: right limbic; RO: right occipital; RP: right parietal; RT: right temporal.

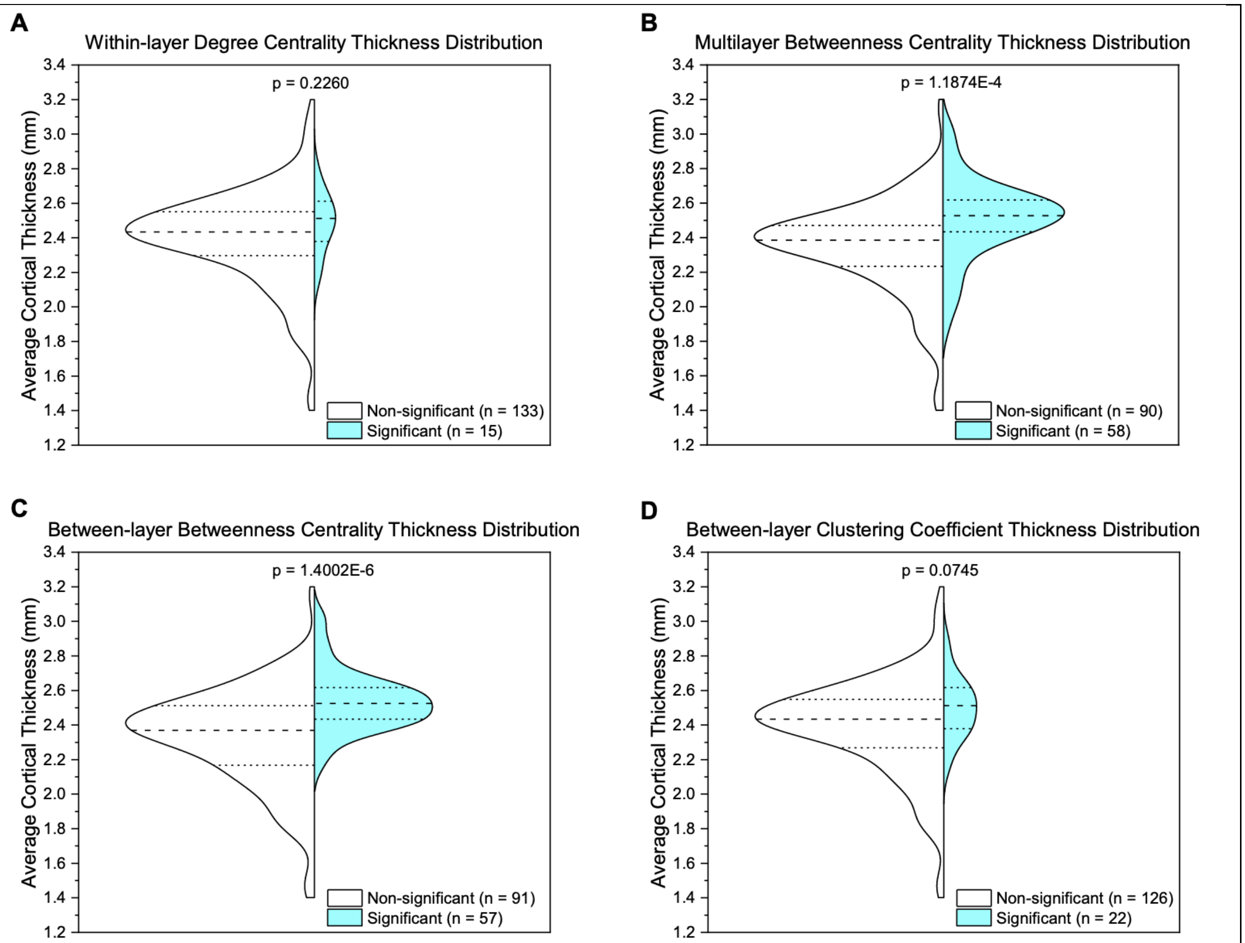

Figure S30. Distribution of thickness values for non-significant versus significant nodes for methods with greater than ten significant nodes: **(A)** within-layer degree centrality, **(B)** multilayer betweenness centrality, **(C)** between-layer betweenness centrality, and **(D)** between-layer clustering coefficient. The white distribution plot (left side) represents non-significant nodes while the teal distribution plot (right side) represents significant nodes. Significance was calculated using a t-test [MATLAB-ttest2].

References

Destrieux, C., Fischl, B., Dale, A., & Halgren, E. (2010). Automatic parcellation of human cortical gyri and sulci using standard anatomical nomenclature. *Neuroimage*, *53*(1), 1-15. https://doi.org/10.1016/j.neuroimage.2010.06.010

Fischl, B., van der Kouwe, A., Destrieux, C., Halgren, E., Segonne, F., Salat, D. H., Busa, E., Seidman, L. J., Goldstein, J., Kennedy, D., Caviness, V., Makris, N., Rosen, B., & Dale, A. M. (2004). Automatically parcellating the human cerebral cortex. *Cereb Cortex*, *14*(1), 11-22. https://doi.org/10.1093/cercor/bhg087

Groppe, D. (2023). *Bonferroni-Holm Correction for Multiple Comparisons*. In (Version 1.1.0.0.) https://www.mathworks.com/matlabcentral/fileexchange/28303-bonferroni-holm-correction-for-multiple-comparisons

Holm, S. (1979). A Simple Sequentially Rejective Multiple Test Procedure. *Scandinavian Journal of Statistics*, *6*(2), 65-70. https://www.jstor.org/stable/4615733
